# The membrane-associated ubiquitin ligase MARCHF8 degrades MHC-I in HPV-positive head and neck cancer for immune evasion

**DOI:** 10.1073/pnas.2525730123

**Published:** 2026-03-09

**Authors:** Mohamed I. Khalil, Jie Wang, Lexi Vu, Canchai Yang, Congcong Yin, Smriti Chadha, Harrison Nabors, Daniel Vocelle, Danielle G. May, Rachel J. Chrisopulos, Craig C. Welbon, Li Zhou, Kyle J. Roux, William C. Spanos, Matthew P. Bernard, Qing-Sheng Mi, Dohun Pyeon

**Affiliations:** ^a^Department of Microbiology, Genetics, and Immunology, Michigan State University, East Lansing, MI 48824; ^b^Department of Molecular Biology, National Research Centre, Cairo 12622, Egypt; ^c^Center for Cutaneous Biology and Immunology Research, Department of Dermatology, Henry Ford Health, Detroit, MI 48202; ^d^Immunology Research Program, Henry Ford Cancer Institute, Henry Ford Health, Detroit, MI 48202; ^e^Department of Medicine, Michigan State University, East Lansing, MI 48824; ^f^Henry Ford Health + Michigan State University Health Sciences, East Lansing, MI 48824; ^g^Department of Pharmacology and Toxicology, Michigan State University, East Lansing, MI 48824; ^h^Enabling Technologies, Sanford Research, Sioux Falls, SD 57104; ^i^Cancer Biology and Immunotherapies Group, Sanford Research, Sioux Falls, SD 57104; ^j^Department of Medicine, Henry Ford Health, Detroit, MI 48202

**Keywords:** human papillomavirus, head and neck cancer, MHC-I, MARCHF8, immune evasion

## Abstract

The loss of major histocompatibility complex class I (MHC-I) molecules has been proposed as a mechanism for cancer immune evasion. Nevertheless, the mechanism is poorly understood. We report here that membrane-associated RING-CH-type finger 8 (MARCHF8), upregulated by human papillomavirus (HPV), ubiquitinates and degrades MHC-I in HPV-positive head and neck cancer (HPV+ HNC). Inhibiting MARCHF8 restores MHC-I levels on HPV+ HNC cells, suppresses tumor growth, and increases the infiltration of natural killer (NK) and T cells in the tumor microenvironment. Furthermore, *Marchf8* knockout markedly increases cross talk between cytotoxic NK cells and CD8^+^ T cells with macrophages and enhances the tumor-killing activity of CD8^+^ T cells. Interestingly, *Marchf8* knockout, in combination with anti-PD-1 treatment, further enhances tumor suppression and increases NK and T cell infiltration in mice bearing immune checkpoint inhibitor–refractory tumors. Our findings suggest that MARCHF8 could be a promising target for immunotherapy for HPV+ HNC patients.

## Significance

Immune checkpoint inhibitors (ICIs) have transformed cancer therapy, yet many human papillomavirus (HPV)-positive head and neck cancers remain resistant due to the downregulation of antigen presentation mechanisms. We identify the ubiquitin ligase MARCHF8 as a primary driver of this evasion that degrades major histocompatibility complex class I (MHC-I) molecules. We demonstrate that *Marchf8* knockout restores antigen presentation, enhances cytotoxic CD8^+^ T cell function, and remodels the tumor microenvironment toward an immunostimulatory state. Importantly, MARCHF8 knockout sensitizes ICI-refractory tumors, highlighting MARCHF8 as a compelling therapeutic target to overcome immunotherapy resistance in HPV-associated head and neck cancers.

Although immune checkpoint inhibitor (ICI) therapy shows promise in treating various cancers, a majority of cancer patients do not respond (1–3). Some solid cancers, including pancreatic, prostate, and head and neck cancers (HNCs), are highly resistant to ICI therapy (1, 4, 5). Human papillomavirus (HPV)-positive HNC (HPV+ HNC) incidence has been dramatically increasing recently despite the effective prophylactic HPV vaccines (6–8). It has been found that the levels of viral epitopes in HPV+ HNC patients are associated with a better response to ICI therapy (9, 10). However, two phase III trials have shown that the response rates to ICI therapies were either similar between HPV+ and HPV-negative (HPV−) HNC patients or even worse in HPV+ HNC patients (1, 11, 12). ICI-refractory patients have limited CD8^+^ T cell infiltration into tumors and low expression of major histocompatibility complex class I (MHC-I) molecules on tumor cells (13–16). Many cancers, including HPV+ HNC, show the downregulation of MHC-I and costimulatory molecules to avoid eliciting T cell responses (17–20). The levels of MHC-I in HPV+ HNC cells are consistently lower than in normal cells (21–24). Nevertheless, the mechanisms of MHC-I downregulation in HPV+ HNC are largely unknown.

Membrane-associated RING-CH-type finger 8 (MARCHF8), an E3 ubiquitin ligase, was initially identified as a human homolog of viral K3 and K5 ubiquitin ligases encoded by Kaposi’s sarcoma-associated herpesvirus (KSHV) (25). Similar to KSHV K3 and K5, MARCHF8 has been found to facilitate evasion of host immune responses (26–28) by ubiquitinating and degrading several immunoreceptors, such as the major histocompatibility complex II (MHC-II) (29) and CD86 (30, 31). MARCHF8 expression is frequently upregulated in esophageal, colorectal, hepatic, and gastric cancer and is associated with tumor progression (32–35). We have recently shown that MARCHF8 is significantly upregulated in HPV+ HNC cells through the HPV oncoprotein-mediated transcriptional activation (36). Further, MARCHF8 degrades death receptors to inhibit cancer cell apoptosis (36) and degrades CUL1 and UBE2L3 to stabilize the HPV oncoprotein E7 (37). These results suggest that MARCHF8 is a potent tumor promoter in HPV+ HNC.

Here, we report that MARCHF8 ubiquitinates and degrades MHC-I proteins and that *Marchf8* knockout suppresses in vivo tumor growth by recruiting and activating CD8^+^ T cells in tumor tissues. *Marchf8* knockout, in combination with an anti-PD-1 inhibitor, significantly improves the survival of mice with ICI-refractory HPV+ HNC. These findings provide an insight into virus-induced cancer immune evasion and a promising therapeutic target to treat ICI-refractory HPV+ HNC patients.

## Results

### MARCHF8 Interacts with MHC-I in HPV+ HNC Cells.

**Fig. 1.**
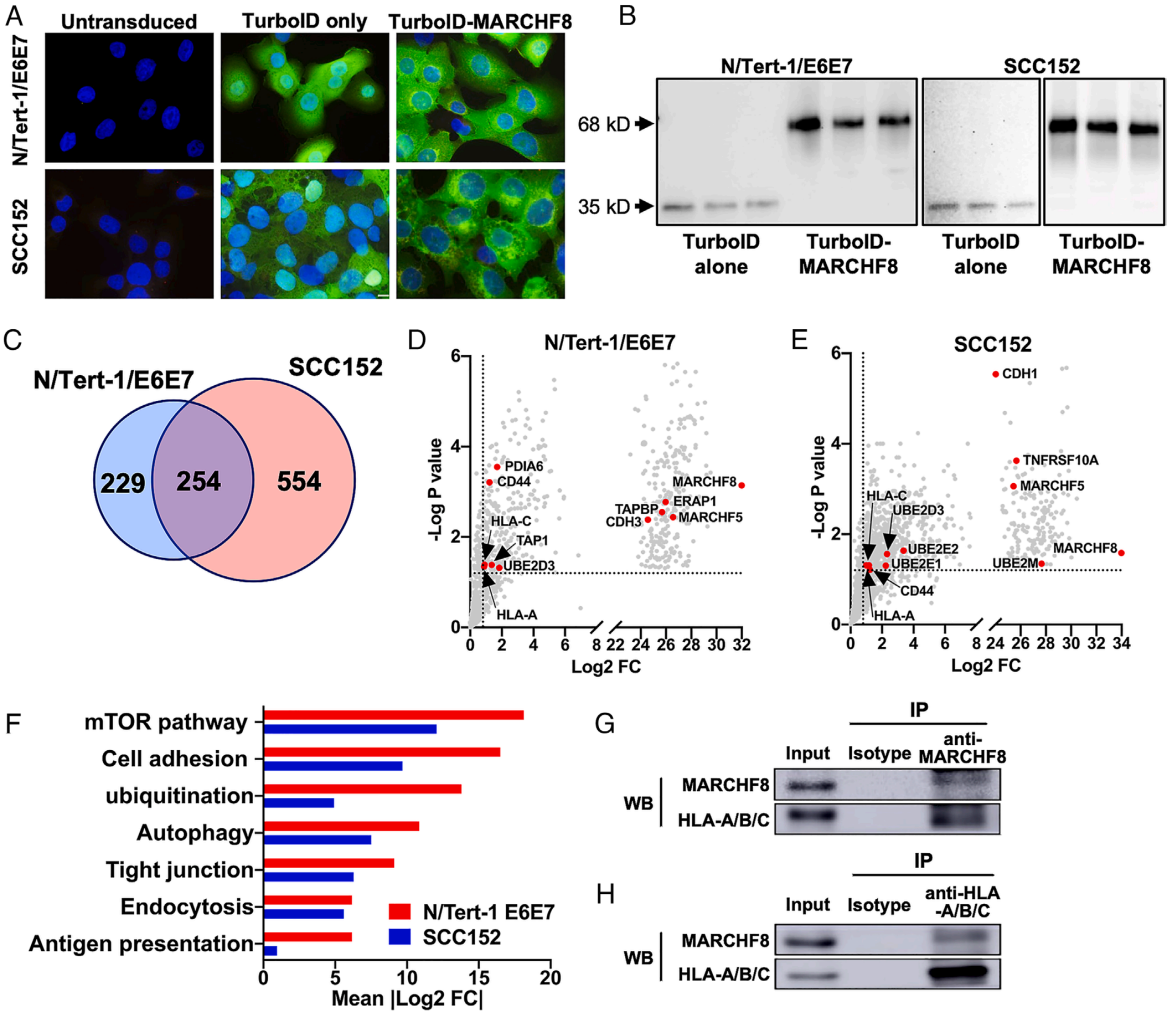
MARCHF8 protein interacts with MHC-I proteins. MARCHF8-TurboID fusion proteins or TurboID proteins alone were stably expressed in N/Tert-1 cells expressing HPV16 oncoproteins E6 and E7 (N/Tert-1/E6E7) and HPV+ HNC cells (SCC152). The TurboID biotinylates the proteins within close proximity. The biotinylated proteins in the cells were separated using streptavidin-coated beads and analyzed using mass spectrometry. Biotinylation by TurboID or TurboID-MARCHF8 was validated by fluorescence microscopy with streptavidin-488 (green) and Hoechst dye (blue) to label DNA (*A*). Expression of TurboID or TurboID-MARCHF8 was validated by western blot with anti-HA (*B*). (*C*) A total of 1,037 proteins enriched with more than a twofold increase and *P*-value less than 0.05 with MARCHF8-TurboID compared to TurboID control in both N/Tert-1 E6E7 (*D*) and SCC152 cells (*E*) were defined as potential MARCHF8 binding proteins. The top pathways involved in MARCHF8 interactions were analyzed by Ingenuity Pathway Analysis (*F*). MARCHF8 (*G*) and HLA-A/B/C (*H*) were pulled down from the cell lysate of SCC152 cells treated with MG132 using anti-MARCHF8 (*G*) and anti-HLA-A/B/C (*H*) antibodies, respectively, and analyzed by western blotting. All experiments were repeated at least three times.

To globally identify the substrates of MARCHF8, we screened MARCHF8 binding proteins in normal immortalized keratinocytes with HPV16 E6 and E7 (N/Tert-1 E6E7) and HPV+ HNC cells (SCC152) using TurboID-based proximity labeling (36). First, stable cell lines expressing TurboID-MARCHF8 fusion protein or TurboID alone were established and validated by immunofluorescence microscopy (Fig. 1*A*) and western blotting (Fig. 1*B*). Then, biotinylated proteins were pulled down in triplicate and analyzed by mass spectrometry. MARCHF8 interacting proteins were defined with proteins that were pulled down with TurboID-MARCHF8 protein significantly higher (>twofold, *P* < 0.05) than TurboID alone in both cell lines (Fig. 1 *C*–*E* and Dataset S1). The validity of the screens was supported by the identification of known immune receptors targeted by MARCHF8 such as CDH1, CD44, and TNF-related apoptosis-inducing ligand receptor 1 (TRAIL-R1) (29, 31, 36) (Fig. 1 *D* and *E*), and enrichment of proteins in the ubiquitination pathway (Fig. 1*F*). Interestingly, analysis using Ingenuity Pathway Analysis software also identified antigen presentation among the top enriched pathways. Further, two MHC-Iα chains, HLA-A and -C, were identified as MARCHF8 interacting proteins in N/Tert-1 E6E7 and SCC152 cells (Fig. 1 *D* and *E*). As MHC-I plays a critical role in the antitumor immune response, we validated the interaction between MARCHF8 and MHC-I proteins. We pulled down MARCHF8 protein in whole cell lysates from SCC152 cells treated with the proteasome inhibitor MG132 using magnetic beads conjugated with an anti-MARCHF8 antibody. The western blot analyses detected HLA-A/B/C protein in the MARCHF8 protein complex pulled down with an anti-MARCHF8 antibody (Fig. 1*G*). Reciprocally, the coimmunoprecipitation of HLA-A/B/C proteins in the same whole cell lysates using an anti-HLA-A/B/C antibody pulled down MARCHF8 protein (Fig. 1*H*), confirming the physical interaction between MARCHF8 and MHC-I proteins.

### Cell Surface Expression of MHC-I on HPV+ HNC Cells Is Downregulated.

**Fig. 2.**
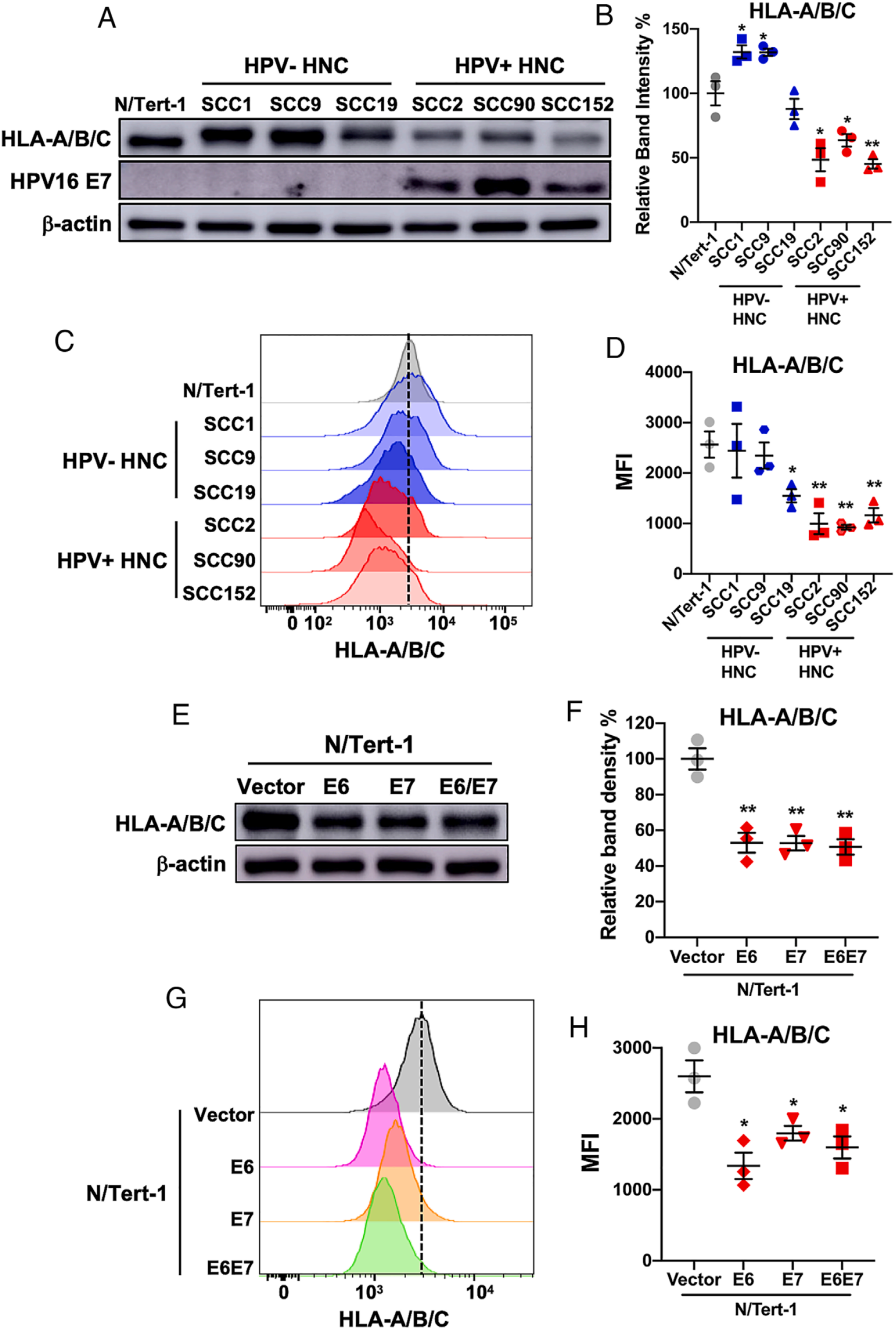
MHC-I expression is downregulated in HPV+ HNC cells. Total protein levels of HLA-A/B/C in HPV-negative (HPV−; SCC1, SCC9, and SCC19) and HPV-positive (HPV+; SCC2, SCC90, and SCC152) HNC cells were determined by western blotting (*A*). Relative band density was quantified using NIH ImageJ (*B*). HPV16 E7 and β-actin were used as viral and cellular controls, respectively. Cell surface expression of HLA-A/B/C (*C* and *D*) on HPV− (SCC1, SCC9, and SCC19) and HPV+ (SCC2, SCC90, and SCC152) HNC cells was analyzed by flow cytometry. Total and cell-surface protein levels of HLA-A/B/C in N/Tert-1 cells expressing HPV16 E6, E7, and E6E7 were determined and compared with the vector-only control by western blotting (*E* and *F*) and flow cytometry (*G* and *H*), respectively. Relative band density was quantified using NIH ImageJ (*F*). Mean fluorescence intensities (MFI) of three independent experiments (*G* and *H*) are shown. All experiments were repeated at least three times, and the data shown are means ± SD. *P* values were determined in pairwise comparisons of each indicated cell line to the N/Tert-1 control cells by Student’s *t* test. *P < 0.05, **P < 0.01.

As MARCHF8 ubiquitinates proteins for degradation, to determine if MHC-I protein levels on HNC cells, we measured protein levels of pan HLA-A/B/C in HPV+ HNC (SCC2, SCC90, and SCC152) and HPV- HNC (SCC1, SCC9, and SCC19) cells compared to normal immortalized keratinocytes (N/Tert-1) cells by western blotting. The results showed that HLA-A/B/C protein levels were significantly lower in HPV+ HNC cells than in N/Tert-1 and HPV− HNC cells (Fig. 2 *A* and *B*). Consistent with the total protein levels, HLA-A/B/C proteins on the surface of all HPV+ HNC cells were significantly decreased compared to N/Tert-1 cells (Fig. 2 *C* and *D*). To further determine if the HPV oncoproteins E6 and/or E7 are sufficient to decrease MHC-I protein levels, we measured HLA-A/B/C protein levels on N/Tert-1 cells expressing HPV16 E6 (N/Tert-1 E6), HPV16 E7 (N/Tert-1 E7), or both HPV16 E6 and E7 (N/Tert-1 E6E7) (37). Interestingly, all N/Tert-1 cells expressing HPV16 E6, E7, or E6E7 significantly decrease MHC-I protein levels in N/Tert-1 cells (Fig. 2 *E*–*H*). These results suggest that MHC-I is downregulated via an HPV16 E6- or E7-driven mechanism and that the regulation is likely at the posttranscriptional level.

### MARCHF8 Knockdown Increases MHC-I Expression.

**Fig. 3.**
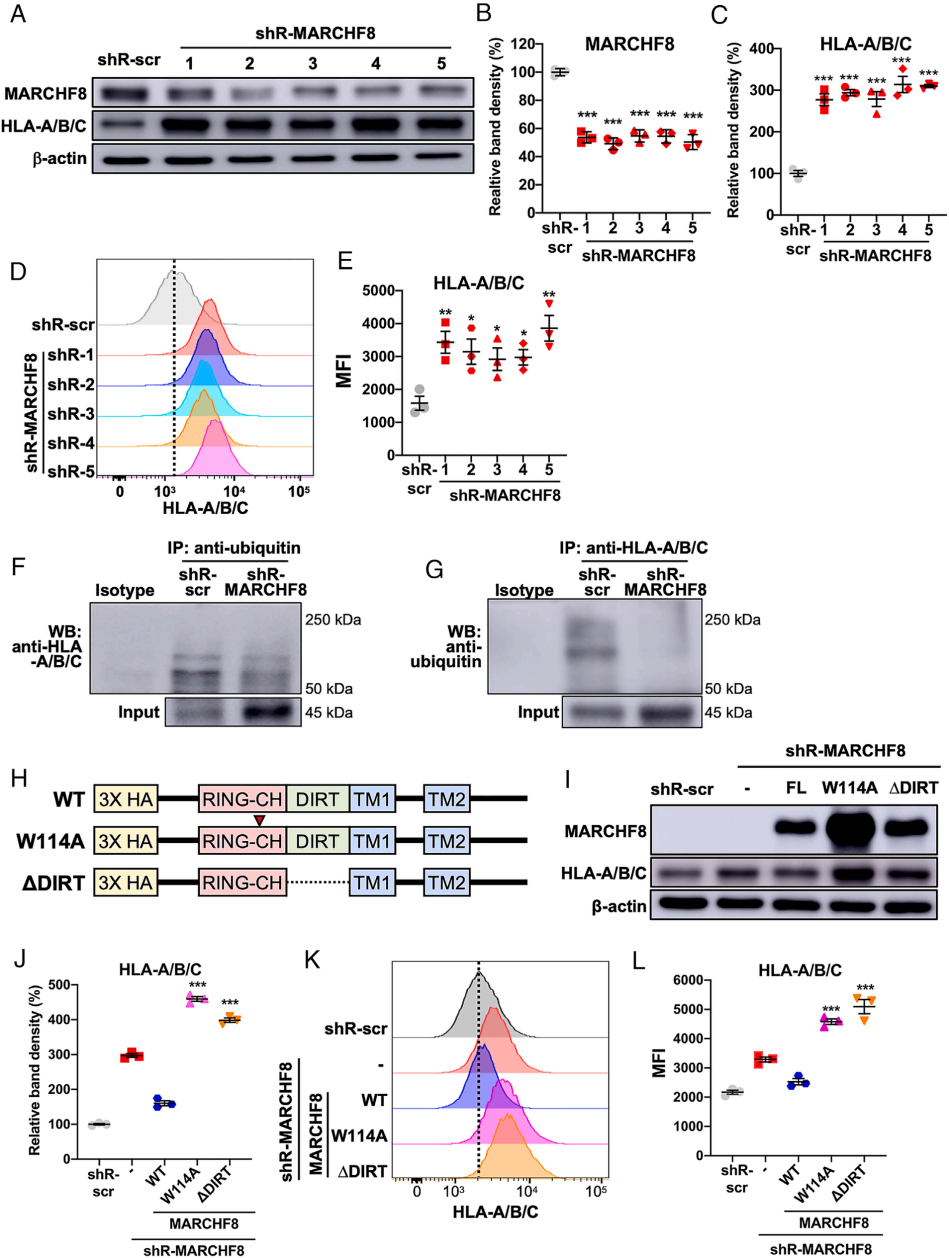
MARCHF8 reduces MHC-I protein levels in HPV+ HNC cells. HPV+ HNC cells (SCC152) were transduced with five lentiviral shRNAs against MARCHF8 (shR-MARCHF8) or shR-scr. MARCHF8 and HLA-A/B/C protein levels were determined by western blotting (*A*–*C*). Relative band density was quantified using NIH ImageJ (*B* and *C*). Cell surface expression of HLA-A/B/C was analyzed by flow cytometry (*D* and *E*). MFIs of three independent experiments (*E*) are shown. Ubiquitinated proteins were pulled down from the lysate of SCC152 cells with shR-scr or shR-MARCHF8 (clone 5) using an antiubiquitin antibody (*F*) and an anti-HLA-A/B/C antibody (*G*) and analyzed by western blotting. HA-tagged WT or mutant MARCHF8 constructs were generated with an amino acid substitution in the RING-CH domain (W114A) and deletion of domain in-between RING and transmembrane (∆DIRT) (*H*). SCC152 cells with shR-MARCHF8 were stably transduced with HA-tagged MARCHF8. MARCHF8 protein expression was determined by western blot using an anti-HA antibody (*I*). Total (*I* and *J*) and cell-surface (*K* and *L*) MHC-I levels were measured by western blot and flow cytometry using anti-HLA-A/B/C antibodies. Relative band density was quantified using NIH ImageJ (*I*). The dotted lines are MFI for control (*D* and *K*). The data shown are means ± SD of three independent experiments. *P* values were determined by Student’s *t* test. *P < 0.05, **P < 0.01, ***P < 0.001.

To determine if MARCHF8 binding to and ubiquitinating MHC-I is responsible for the decrease of MHC-I protein levels in HPV+ HNC cells, we knocked down MARCHF8 expression in HPV+ HNC cells (SCC152 and SCC2) using multiple shRNAs against MARCHF8 (shR-MARCHF8) delivered by lentiviruses and selected by puromycin treatment. All SCC152 (Fig. 3 *A* and *B*) and SCC2 (*SI Appendix*, Fig. S1 *A* and *B*) cell lines with shR-MARCHF8 showed at least a 50% decrease in total MARCHF8 protein levels compared to the control cell lines with scrambled shRNA (shR-scr). Next, we analyzed MHC-I protein levels by western blotting and flow cytometry using an anti-pan-HLA-A/B/C antibody. We found that total (Fig. 3 *A* and *C* and *SI Appendix*, Fig. S1 *A* and *C*) and cell surface (Fig. 3 *D* and *E* and*SI Appendix*, Fig. S1 *D* and *E*) protein levels of HLA-A/B/C are significantly increased by MARCHF8 knockdown. To determine whether the knockdown of MARCHF8 expression affects mRNA expression of MHC-I, we measured mRNA levels of HLA-A, HLA-B, and HLA-C in the SCC152 and SCC2 cell lines by RT-qPCR and found that none of the SCC152 and SCC2 cells showed an increase in MHC-I mRNA levels by MARCHF8 knockdown (*SI Appendix*, Fig. S2). These results suggest that MARCHF8 downregulation of MHC-I proteins likely occurs posttranscriptionally.

### MARCHF8 Knockdown Decreases MHC-I Ubiquitination.

To determine whether MARCHF8 induces ubiquitination of MHC-I proteins, we pulled down ubiquitinated proteins from whole-cell lysates of SCC152 cells treated with MG132 using magnetic beads conjugated with an antiubiquitin antibody. The results showed that the levels of ubiquitinated HLA-A/B/C proteins were decreased by MARCHF8 knockdown, despite the significantly higher levels of total input proteins of HLA-A/B/C, compared to SCC152 cells with shR-scr (Fig. 3*F*). We reciprocally pulled down HLA-A/B/C proteins from the same SCC152 cell lysates using an anti-HLA-A/B/C antibody and detected ubiquitinated HLA-A/B/C proteins with an antiubiquitin antibody. Consistently, the levels of ubiquitinated HLA-A/B/C proteins in SCC152 cells with MARCHF8 knockdown are significantly reduced compared to those in SCC152 cells with scrambled shRNA (Fig. 3*G*). As the majority of ubiquitinated proteins are degraded via the proteasome, HLA-A/B/C protein levels were measured in SCC152 cells treated with the proteasome inhibitor MG132 using western blotting (*SI Appendix*, Fig. S3 *A* and *B*) and flow cytometry (*SI Appendix*, Fig. S3 *C* and *D*). Surprisingly, the HLA-A/B/C proteins were not accumulated by MG132 treatment (*SI Appendix*, Fig. S3 *A*–*D*). Our findings suggest that MHC-I protein expression in HPV+ HNC cells is likely reduced by MARCHF8-mediated MHC-I ubiquitination via a proteasome-independent pathway.

Next, we determined whether IFN-γ treatment upregulates MHC-I expression in HPV+ HNC cells with MARCHF8 knockdown. We treated SCC152 with shR-scr and shR-MARCHF8 (clone 5) in the presence of 1 ng/mL of IFN-γ for 16 h and determined HLA-A/B/C protein levels by western blotting (*SI Appendix*, Fig. S4*A*) and flow cytometry (*SI Appendix*, Fig. S4*C*), respectively. Activation of IFN-γ signaling was validated by measuring phospho-STAT1 (pSTAT1) levels (*SI Appendix*, Fig. S4*A*). While IFN-γ treatment significantly increased HLA-A/B/C levels in both SCC152 cells with and without MARCHF8 knockdown, MARCHF8 knockdown further increased HLA-A/B/C protein levels, as shown in Fig. 3 (*SI Appendix*, Fig. S4*B*). However, cell surface HLA-A/B/C levels did not increase by MARCHF8 knockdown (*SI Appendix*, Fig. S4*D*). These findings indicate that treatment with IFN-γ significantly enhances MHC-I expression to peak levels, and degradation mediated by MARCHF8 has a minimal impact on MHC-I levels.

### Expression of Wild-Type (WT) MARCHF8, but Not Mutant MARCHF8, Decreases MHC-I Levels.

To determine whether MARCHF8 ubiquitin ligase activity is required for MHC-I downregulation, generated SCC152 cell lines with MARCHF8 knockdown that express HA-tagged WT MARCHF8 or MARCHF8 mutants that have an amino acid substitution in the RING-CH domain (W114A) or a deletion of the domain in-between RING and transmembrane (∆DIRT) (Fig. 3*H*). Consistent with our results above, the expression of WT MARCHF8 significantly decreased the total (Fig. 3 *I* and *J*) and cell surface (Fig. 3 *K* and *L*) MHC-I levels. In contrast, the expression of the MARCHF8 W114A and MARCHF8 ΔDIRT did not decrease the total (Fig. 3 *I* and *J*) and cell surface MHC-I (Fig. 3 *K* and *L*) levels in SCC152 cells with MARCHF8 knockdown. These results suggest that the ubiquitination function of MARCHF8 is required to decrease MHC-I protein levels.

### *Marchf8* Knockout in Syngeneic Mouse HPV+ HNC Cells Increases MHC-I Levels and Sensitizes Tumors to Anti-PD-1 Immunotherapy.

**Fig. 4.**
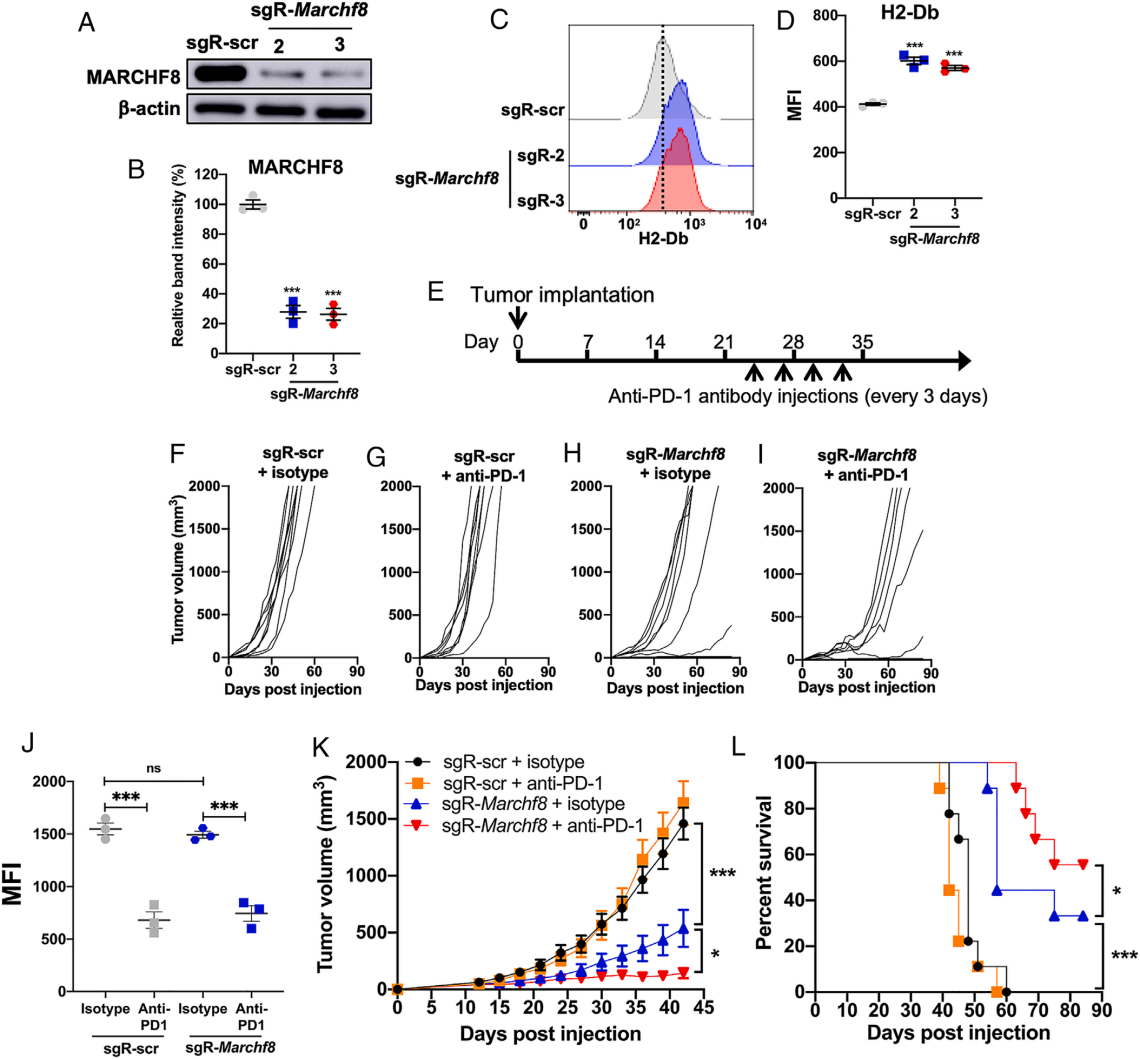
*Marchf8* knockout enhances antitumor activity in combination with anti-PD-1 treatment. mEERL cells were transduced with lentiviral *Cas9* and one of two sgRNAs against *Marchf8* (sgR-*Marchf8-2* and sgR-*Marchf8-3*) or sgR-scr. MARCHF8 protein levels were determined by western blotting (*A*), and relative band density was quantified using NIH ImageJ (*B*). The data shown are means ± SD of three independent experiments. Cell surface levels of H2-Db protein were measured by flow cytometry (*C* and *D*). The dotted line is MFI for control (*C*). MFI of three independent experiments is shown (*D*). *P* values were determined by Student’s *t* test. ***P < 0.001. C57BL/6J mice (*n* = 9 per group) with mEERL/scr or mEERL/*Marchf8*^−/−^ cells were injected into the rear right flank with four doses (200 μg each) of either rIgG2a isotype or anti-PD-1 antibodies (clone RMP1-14), starting from day 24 (*E*). Tumor volume was measured twice a week (*F*–*K*). Dot plot showing the MFI values for PD1 expression on the surface of CD8^+^ T cells isolated from the TME of mice injected with mEERL sgR-scr and mEERL sgR-Marchf8-2 treated with anti-PD1 or isotype control antibody (*J*). Survival rates of mice were analyzed using a Kaplan–Meier estimator (*L*). The time to event was determined for each group, with the event defined as a tumor size of 2,000 mm^3^. The data shown are means ± SD. *P* values of mice injected with mEERL/*Marchf8*^−/−^ cells compared with mice injected with mEERL/scr cells were determined for tumor growth (*K*) and survival (*L*) by two-way ANOVA analysis. Shown are representative of two independent experiments.

To assess whether *Marchf8* knockout suppresses MHC-I expression in mice, we used an immunocompetent syngeneic mouse model of mEERL cells expressing HPV16 E6 and E7 along with *Hras*. We first established *Marchf8* knockout mEERL (mEERL/*Marchf8*^−/−^) cell lines using lentiviral transduction of *Cas9* and one of two small guide RNAs (sgRNAs) against *Marchf8* (sgR-*Marchf8*, clones 2 and 3). mEERL cells transduced with *Cas9* and scrambled sgRNA (sgR-scr) (mEERL/scr) were used as a control. Both mEERL cell lines transduced with sgR-*Marchf8* showed a ~75% decrease in MARCHF8 protein levels compared to mEERL/scr cells (Fig. 4 *A* and *B*). Consistent with the data from human HPV+ HNC cells presented in Fig. 3 *D* and *E*, mEERL/*Marchf8*^−/−^ (sgR-*Marchf8-2* and sgR-*Marchf8-3*) cells showed significantly upregulated H2-Db (MHC-I haplotype in mEERL cells and C57BL/6J mouse) on the cell surface compared to mEERL/scr cells (Fig. 4 *C* and *D*). Next, either mEERL/*Marchf8*^−/−^ cells or mEERL/scr cells were subcutaneously injected into syngeneic C57BL/6J mice. Tumor growth was monitored twice a week for 12 wk. All 10 mice injected with mEERL/scr cells developed vigorous tumor growth (*SI Appendix*, Fig. S5*A*) and succumbed to tumor burden within ~7 wk postinjection (*SI Appendix*, Fig. S5*B*). In contrast, the majority of the mice, each injected with either of two mEERL/*Marchf8*^−/−^ cells, showed significantly delayed or no tumor growth (*SI Appendix*, Fig. S5 *A* and *B*). To validate MARCHF8 expression levels in tumor tissues, MARCHF8 levels in mEERL cells were compared with those in tumor tissues by western blotting (*SI Appendix*, Fig. S5 *C* and *D*). As shown in Fig. 4*A*, MARCHF8 protein levels are reduced by ~75% in tumor tissues from mice injected with mEERL/*Marchf8*^−/−^ cells compared to mEERL/scr cells. These results suggest that MARCHF8 is a potent tumor promoter that plays a vital role in HPV+ HNC tumor growth.

Previous studies have shown that the mEERL mouse model represents the ICI-refractory HPV+ HNCs (38–40). To test if MHC-I increases by *Marchf8* knockout sensitizes mEERL tumors to anti-PD-1 blockade, mice injected with mEERL/*Marchf8^−/−^* or mEERL/scr cells were treated with four doses of an anti-PD-1 (clone #RMP1-14) or IgG2b isotype antibody every 3 d starting from 24 d postinjection, when the average tumor size in mice with mEERL/scr cells was 200 mm^3^ (Fig. 4*E*). Regardless of the treatment with an anti-PD-1 antibody or an IgG2b isotype antibody, all mice with mEERL/scr cells showed vigorous tumor growth (Fig. 4 *F*, *G*, and *J*) and succumbed to tumor burden in about 8 wk postinjection (Fig. 4*K*). In contrast, the mice injected with mEERL/*Marchf8*^−/−^ (sgR-*Marchf8-2*) cells consistently showed significant tumor suppression (Fig. 4*H*). Interestingly, the combination of *Marchf8* knockout and anti-PD-1 antibody treatment further enhances tumor suppression (Fig. 4 *I* and *J*) and improves mouse survival, resulting in three out of nine mice with tumor-free survival (Fig. 4*K*). These results indicate that MHC-I protein degradation plays a key role in HPV+ HNC immune evasion and that combining *Marchf8* knockout with PD-1 inhibition may be an effective treatment for ICI-refractory HPV+ HNCs.

### *Marchf8* Knockout Alters Immune Cell Infiltration in the Tumor Microenvironment of HPV+ HNC.

**Fig. 5.**
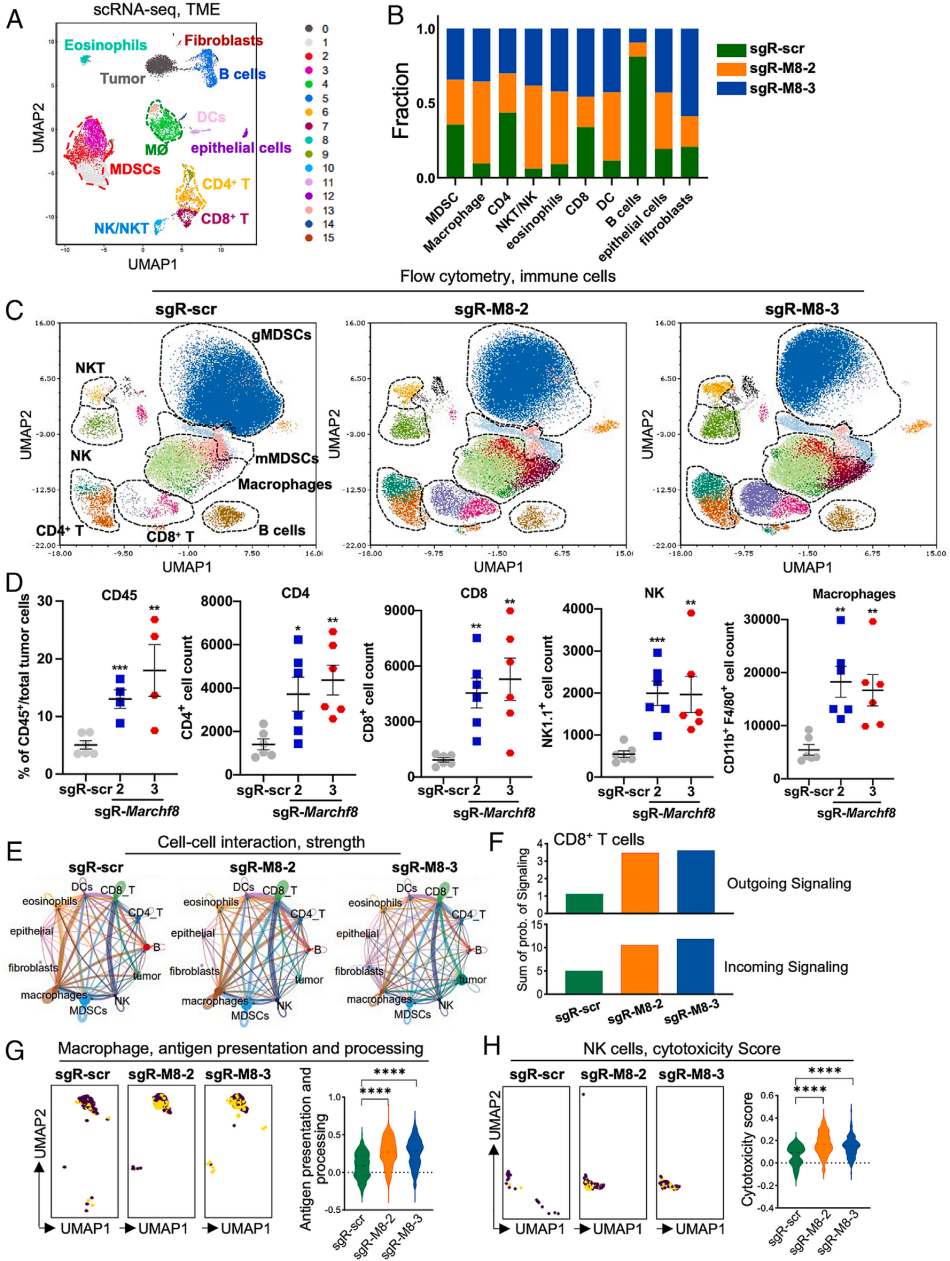
*Marchf8* knockout in HPV+ HNC cells increases NK and CD8^+^ T cells in the TME. Tumors were isolated from C57BL/6J mice injected with mEERL/scr or mEERL/*Marchf8*^−/−^ (sgR-*Marchf8-2* and sgR-*Marchf8-3*) cells. The single cells isolated from the tumor tissues were sequenced for scRNA-seq. The UMAP plots of integrated samples (*A*) and the distribution of each immune cell cluster in *Marchf8* knockout (sgR-Marchf8-2 and sgR-Marchf-3) and sgR-scr are shown in the bar graph (*B*). CD45^+^ immune cells were stained with an antibody cocktail and analyzed by flow cytometry. The UMAP plots of flow cytometry data show the color-coded distributions of infiltrating immune cells as indicated (*C*). Dot plots show the frequency of CD45^+^ cells and the cell counts of CD4^+^ and CD8^+^ T cells, NK cells, and macrophages from flow cytometry analysis (*D*). All flow cytometry experiments were repeated at least three times, and the data shown are means ± SD. *P* values were determined by Student’s *t* test. *P < 0.05, **P < 0.01, ***P < 0.001, ****P < 0.0001. CCI was analyzed among different immune cells, and the strength of the CCI was presented as the thickness of the strings (*E*). Bar graphs show the combined outgoing and incoming signaling of CD8^+^ T cells (*F*). UMAP plots show enrichment of the antigen presentation and processing pathway in macrophages. The yellow dots represent the positive values in the indicated pathway, and the violin plot shows the distribution of antigen presentation and processing scores in macrophages (*G*). UMAP plots show the cytotoxicity enrichment in NK cells; the dots in yellow represent the positive in the indicated pathway, and the violin plot shows the score of the cytotoxicity enrichment in NK cells (*H*).

Given that *Marchf8* knockout potentiates anti-PD-1 immunotherapy, we examined the tumor microenvironment (TME) to further elucidate the mechanism of tumor suppression by *Marchf8* knockout. We performed immune cell profiling in tumor tissues harvested from the C57BL/6J mice injected with mEERL/*Marchf8*^−/−^ or mEERL/scr cells when the average tumor size of the sgR-scr is 1,000 mm^3^ at day 30, using single-cell RNA sequencing (scRNA-seq) and flow cytometry. scRNA-seq of 7,965 cells in mEERL/scr tumors, 5,120 cells in mEERL/*Marchf8*^−/−^ tumors (sgR-Marchf8-2), and 6,402 cells in mEERL/*Marchf8*^−/−^ tumors (sgR-Marchf8-3) identified 12 immune cell populations (Fig. 5 *A* and *B*) (41). The results showed that macrophage (Clusters #4 and #13), NK/NKT cell (Cluster #10), eosinophils (Cluster #8), and dendritic cell (DC) (Cluster #11) populations were increased in mEERL/*Marchf8*^−/−^ tumors (sgR-*Marchf8-2* and sgR-*Marchf8-3*). In contrast, the B cell (Cluster #5) population was decreased (Fig. 5 *A* and *B*). Myeloid-derived suppressor cells (MDSCs, Clusters #1, #2, and #3), CD8^+^ T cells (Cluster #7), and CD4 T cells (Clusters #6 and #9) showed no change or slight increase in mEERL/*Marchf8*^−/−^ tumors. To validate the key immune cell populations at the cellular level, we performed a high-dimensional flow cytometry analysis using a 15-antibody panel (*SI Appendix*, Table S1). Our gating strategy for immune cell phenotyping is shown in *SI Appendix*, Fig. S6. The results showed significant changes in immune cell composition in the TME by *Marchf8* knockout (Fig. 5*C*). Especially, the frequency of CD45^+^ and CD3^+^ cells was dramatically increased in the TME of mEERL/*Marchf8*^−/−^ tumors (sgR-*Marchf8-2* and sgR-*Marchf8-3*) (Fig. 5*D* and *SI Appendix*, Fig. S7 *A*–*C*). Although scRNA-seq data showed no change or slight increase in CD4^+^ T cells and CD8^+^ T cells by *Marchf8* knockout, our flow cytometry analysis revealed a significant increase in CD4^+^ T cell and CD8^+^ T cell populations in both mEERL/*Marchf8*^−/−^ tumors (Fig. 5 *C* and *D* and *SI Appendix*, Fig. S7*D*). This discrepancy could be due to dropout effects in scRNA-seq and to dissociation bias between scRNA-seq and flow cytometry. Additionally, our flow cytometry analysis showed significant increases in NK cell and macrophage infiltration into the TME of mEERL/*Marchf8^−/−^* tumors (sgR-*Marchf8-2* and sgR-*Marchf8-3*) (Fig. 5*D* and *SI Appendix*, Fig. S7 *E* and *I*). Furthermore, while scRNA-seq data showed comparable numbers of MDSCs between mEERL/scr and mEERL/*Marchf8^−/−^* tumors (Fig. 5*B*), we observed that granulocytic MDSC (gMDSC: CD11b^+^MHCII^−^CD11c^−^Ly6G^+^) population was significantly reduced, while monocytic MDSC (mMDSC: CD11b^+^MHCII^−^CD11c^−^Ly6C^+^) population was increased in mEERL/*Marchf8^−/−^* cells compared to the sgR-scr tumors (Fig. 5*C* and *SI Appendix*, Fig. S7 *F*–*H*). These results suggest that the *Marchf8* knockout significantly increases NK cells, T cells, and macrophages, potentially remodeling the TME from an immunosuppressive to an immunostimulatory environment.

To determine how *Marchf8* knockout affects crosstalk among immune cells in the TME, we performed a cell–cell interaction (CCI) analysis using scRNA-seq data. The results suggested that the *Marchf8* knockout significantly enhanced the interactions of CD8^+^ T cells with macrophages and NK cells in mEERL/*Marchf8^−/−^* tumors (sgR-*Marchf8-2* and sgR-*Marchf8-3*) compared to mEERL/scr tumors (Fig. 5*E*). Furthermore, both the outgoing signaling from CD8^+^ T cells and incoming signaling to the CD8^+^ T cells were enhanced by *Marchf8* knockout (Fig. 5*F*). These results suggest that *Marchf8* knockout might induce active cell–cell communications of CD8^+^ T cells with other immune cells in the TME. To delve deeper into how *Marchf8* affects CD8^+^ T cell interactions with macrophages and NK cells, we performed the KEGG pathway analysis on macrophage and NK cell clusters using scRNA-seq data. Interestingly, macrophage antigen presentation and processing pathway (Fig. 5*G*) and the NK cell cytotoxicity scores (Fig. 5*H*) were significantly increased in the mEERL/*Marchf8^−/−^* tumors. These results suggest that the *Marchf8* knockout may enhance the antigen presentation of macrophages, which is crucial for initiating and sustaining an effective immune response. Thus, *Marchf8* knockout may enhance the crosstalk between CD8^+^ T cells and other immune cells in the TME and may also boost the functional capacities of macrophages.

### Anti-PD-1 Antibody Treatment Further Enhances the Immunostimulatory TME Generated by *Marchf8* Knockout in HPV+ HNC Cells.

Next, we tested if anti-PD-1 antibody treatment further enhances the infiltration of antitumor immune cells into the TME. To characterize immune cell infiltration in the TME altered by *Marchf8* knockout in combination with anti-PD-1 antibody, we performed immune cell profiling in tumor tissues from the C57BL/6J mice injected with mEERL/*Marchf8*^−/−^ (sgR-*Marchf8-2*) or mEERL/scr cells with or without anti-PD-1 antibody treatment. Our data showed that anti-PD-1 antibody treatment decreased PD-1 levels on CD8^+^ T cells (Fig. 4*J*) but did not increase the infiltration of CD45^+^ immune cells into tumors from mEERL/scr cells (*SI Appendix*, Fig. S8 *A* and *B*). In contrast, CD45^+^ immune cell infiltration in the TME was highly enhanced by anti-PD-1 antibody in mice injected with mEERL/*Marchf8*^−/−^ cells (*SI Appendix*, Fig. S8 *A* and *B*). Additionally, the anti-PD-1 treatment increased the numbers of CD3^+^ cells in both mEERL/*Marchf8*^−/−^ and mEERL/scr cells (*SI Appendix*, Fig. S8 *C* and *D*). Interestingly, anti-PD-1 treatment increased the numbers of CD4^+^ T cells only in mEERL/scr cells (*SI Appendix*, Fig. S8 *E* and *F*). The number of CD8^+^ T cells increased only in mice injected with mEERL/*Marchf8*^−/−^ cells (*SI Appendix*, Fig. S8 *E* and *F*). Additionally, the number of NK cells, macrophages, and mMDSCs was increased in both mEERL/*Marchf8*^−/−^ and mEERL/scr cells treated with the anti-PD-1 antibody compared with cells treated with the IgG2b control antibody (*SI Appendix*, Fig. S8 *G*–*L*). In contrast, the number of gMDSCs decreased only in the TME of the anti-PD-1 antibody-treated mice injected with mEERL/scr cells (*SI Appendix*, Fig. S8 *I* and *J*). Our results suggest that anti-PD-1 antibody treatment overcomes CD8^+^ T cell exhaustion induced by *Marchf8* knockout and that MARCHF8 could be an effective target in combination with ICI therapy for treating ICI-refractory HPV+ HNC patients.

### *Marchf8* Knockout in HPV+ HNC Cells Induces Tumor Cell–Killing Functions of CD8+ T Cells.

To determine how *Marchf8* knockout impacts CD8^+^ T cell functions in the TME, we assessed the expression of cytotoxicity-associated genes in CD8^+^ T cells altered by *Marchf8* knockout (*SI Appendix*, Fig. S9*A*). Our results showed a significant increase in the cytotoxicity score of CD8^+^ T cells in the TME of mEERL/*Marchf8^−/−^* tumors (sgR-*Marchf8-2* and sgR-*Marchf8-3*) compared to mEERL/scr tumors (*SI Appendix*, Fig. S9*B*). Specifically, CD8^+^ T cells in the *Marchf8* knockout tumors exhibited significantly increased expression of *Gzmb, Gzma, Prf1, CD44, Ifng,* and *Tbx21* (*SI Appendix*, Fig. S9*C*), indicating that *Marchf8* knockout induces CD8^+^ T cell effector functions for tumor cell killing.

To validate these results, we examined CD8^+^ T cell functions, including proliferation, IFN-γ production, and tumor cell killing. First, to analyze CD8^+^ T cell proliferation, CD8^+^ T cells were isolated from mice injected with mEERL/scr (designated as null CD8^+^ T cells) or mEERL/*Marchf8*^−/−^ cells (designated as primed CD8^+^ T cells). The isolated null or primed CD8^+^ T cells were labeled with carboxyfluorescein diacetate succinimidyl ester (CFSE) and cocultured with mitomycin C-treated target cells (mEERL/scr or mEERL/*Marchf8*^−/−^ cells). The results showed that the proliferation of CD8^+^ T cells isolated from mice with mEERL/*Marchf8*^−/−^ cells is significantly enhanced compared to CD8^+^ T cells from mice with mEERL/scr cells (*SI Appendix*, Fig. S9 *D* and *E*). Naïve CD8^+^ T cells without tumor cells (mEERL) in the presence and absence of anti-CD3/CD28 beads were used as negative controls. All controls showed a negligible change in CD8^+^ T cell proliferation in the absence of mEERL cells, even when treated with anti-CD3/CD28 antibody beads (*SI Appendix*, Fig. S9*F*). To determine if *Marchf8* knockout in HPV+ HNC cells activates CD8^+^ T cells, we measured IFN-γ production from CD8^+^ T cells from mEERL/*Marchf8*^−/−^ and mEERL/scr cells using ELISA. The isolated null or primed CD8^+^ T cells were cocultured with mitomycin C-treated target mEERL/*Marchf8*^−/−^ or mEERL/scr cells. CD8^+^ T cells stimulated with phorbol-12-myristate-13-acetate and ionomycin (PMA/Iono) were used as a positive control (*SI Appendix*, Fig. S9*F*). Our results showed that IFN-γ production was significantly increased in primed CD8^+^ T cells cocultured with mEERL/*Marchf8*^−/−^ cells but not in null CD8^+^ T cells cocultured with mEERL/scr cells (*SI Appendix*, Fig. S9*F*). The absence of mEERL cells in the absence of anti-CD3/CD28 beads resulted in a very low level of IFN-γ production by CD8^+^ T cells (*SI Appendix*, Fig. S9*F*). Next, to assess the cytolytic capacity of CD8^+^ T cells affected by *Marchf8* knockout in HPV+ HNC cells, we performed a lactate dehydrogenase (LDH) release assay that measures LDH in the cell culture supernatant released by cell lysis. After the isolated null or primed CD8^+^ T cells were incubated with mEERL/scr or mEERL/*Marchf8*^−/−^ cells for 24 h, the cell culture supernatant was collected, and LDH levels were measured by an enzymatic coupling reaction and absorbance. Interestingly, primed CD8^+^ T cells showed significantly enhanced killing of mEERL/*Marchf8*^−/−^ cells compared to null CD8^+^ T cell killing of mEERL/scr cells (*SI Appendix*, Fig. S9*G*), suggesting that *Marchf8* knockout significantly enhances the antitumor activity of CD8^+^ T cells. We assessed the abilities of the primed CD8^+^ T cells to kill normal immortalized mouse oral epithelial cells (NIMOE). The ability of the primed CD8^+^ T cells to kill the NIMOE cells is comparable to mEERL/scr cells (*SI Appendix*, Fig. S9*G*). Our findings suggest that targeting MARCHF8 restores MHC-I expression on HPV+ HNC cells, activates effector CD8^+^ T cells, and suppresses tumor growth (*SI Appendix*, Fig. S9*H*).

### CD8^+^ T Cells Are Required for Significant Tumor Suppression by *Marchf8* Knockout in HPV+ HNC Cells.

Given that *Marchf8* knockout upregulates MHC-I in HPV+ HNC cells and activates CD8^+^ T cells for tumor cell killing (Figs. 4 and 5 and *SI Appendix*, Fig. S6), we hypothesized that CD8^+^ T cells are required for tumor suppression by targeting MARCHF8. To test the hypothesis, CD8^+^ T cells were depleted in C57BL/6J mice using an anti-CD8α neutralizing antibody (*SI Appendix*, Fig. S10*A*), as described previously (21). Ten doses of an anti-CD8α neutralizing antibody or an IgG2a isotype antibody were intraperitoneally injected into mice with mEERL/*Marchf8*^−/−^ tumors. To validate CD8^+^ T cell depletion in mice, the number of CD8^+^ T cells in splenocytes was counted using an anti-CD8α antibody (clone 2.43) specific to epitopes distinct from those of the anti-CD8α neutralizing antibody used for depletion (*SI Appendix*, Table S1). The results showed that approximately 90% of CD8^+^ T cells were depleted (*SI Appendix*, Fig. S10*B*). Next, we monitored tumor growth and found that all CD8^+^ T cell–depleted mice injected with mEERL/*Marchf8*^−/−^ cells showed vigorous tumor growth (*SI Appendix*, Fig. S10 *C* and *D*) and succumbed to tumor burden in about 7 wk postinjection, albeit slightly delayed (*SI Appendix*, Fig. S10*E*), despite *Marchf8* knockout. In contrast, *Marchf8* knockout significantly suppresses tumor growth (*SI Appendix*, Fig. S10 *C* and *D*) and extended survival (*SI Appendix*, Fig. S10*E*) of the isotype control mice injected with mEERL/*Marchf8*^−/−^ cells compared to the mice injected with mEERL/scr. These results suggest that although MARCHF8 targets several surface immune receptors, the dominant driver of tumor suppression by *Marchf8* knockout is the restoration of MHC-I-dependent CD8^+^ T cell–mediated killing.

## Discussion

MHC-I molecules are among the most common targets for immune evasion in cancers due to their vital role in antitumor CD8^+^ T cell responses by presenting antigens (22, 42–47). Downregulation of MHC-I impairs antitumor immune responses and attenuates immunotherapies that reactivate antitumor CD8^+^ T cells by ICIs (38, 39). Thus, cancer cells develop various mechanisms to hinder MHC-I antigen presentation, and a better understanding of how cancer cells dysregulate MHC-I antigen presentation is critical.

Unlike herpesviruses, HPV does not encode E3 ubiquitin ligases, but its viral oncoproteins E6 and E7 hijack host E3 ubiquitin ligases to target host proteins, thereby evading the host immune response (48, 49). We have recently discovered that the HPV oncoproteins upregulate the expression of MARCHF8, a membrane-associated ubiquitin ligase homologous to KSHV K3 and K5, by activating the MYC/MAX transcription factor complex (25–27, 36, 50). MARCHF8, a member of the MARCHF E3 ubiquitin ligase family, localizes in the plasma and endosomal membranes (51), and degrades various membrane proteins (30, 31, 52). We have recently revealed that MARCHF8 ubiquitinates and degrades the death receptors from the TNF receptor superfamily (36). Additionally, we report here that MARCHF8 ubiquitinates and degrades MHC-I and that MARCHF8 knockout induces antitumor immune responses by restoring MHC-I expression and activating CD8^+^ T cells in the TME of HPV+ HNC.

In this study, *Marchf8* knockout with an anti-PD-1 antibody treatment dramatically suppresses ICI-refractory HPV+ HNC tumor growth. Thus, the development of MARCHF8 inhibitors could improve upon ICIs. Here, we show not only that *Marchf8* knockout increases MHC-I and inhibits tumor growth but also that the number of cytotoxic effector CD8^+^ T cells highly increased in the TME. Mice with depleted CD8^+^ T cells showed vigorous tumor growth even with *Marchf8* knockout. These results suggest that the observed tumor suppression by *Marchf8* knockout is primarily driven by CD8^+^ T cells. However, tumor growth in *Marchf8* knockout mice was still suppressed by CD8^+^ T cell depletion compared to control tumors. While incomplete depletion of CD8^+^ T cells may partially explain this discrepancy, other immune cells in the TME can also contribute to tumor cell killing. Indeed, both single-cell RNA seq and flow cytometry analyses showed that the number of NK cells in the TME significantly increased following *Marchf8* knockout. Future studies depleting NK cells and monitoring tumor growth following injection with HPV+ HNC cells with *Marchf8* knockout will help delineate the roles of NK vs. CD8^+^ T cells in tumor suppression by *Marchf8* knockout. Another possibility for suppressed tumor growth even in the absence of CD8^+^ T cells may be caused by additional tumor suppressors targeted by MARCHF8. We have previously shown that knocking down MARCHF8 significantly increases the rate of apoptosis in HPV+ HNC cells by upregulating death receptors (36). Additionally, by targeting the E3 ubiquitin ligase CUL1 and associated E2 UBE2L3, MARCHF8 stabilizes the HPV E7 oncoprotein and promotes cell cycle progression, a phenotype reversed by MARCHF8 knockdown (37). Therefore, tumor suppression by *Marchf8* knockout involves multiple mechanisms, including enhanced CD8^+^ T cell responses through restored antigen presentation, increased apoptosis via elevated death receptors, and destabilization of the E7 viral oncoprotein. Although increased MHC-I expression following MARCHF8 knockout provides a compelling mechanistic link to enhanced CD8^+^ T cell–mediated immunity, our data do not exclude contributions from additional MARCHF8-regulated pathways. Given that MARCHF8 is an E3 ubiquitin ligase with multiple potential substrates, it is likely that loss of MARCHF8 affects immune responses through both MHC-I-dependent and -independent mechanisms.

FDA-approved ICI therapy has shown promising results in treating patients with previously untreatable cancers (53, 54). However, clinical trials have shown that most HNC patients do not respond to ICI therapy (1, 55). Recent studies to better understand the mechanisms by which cancer cells develop resistance to ICIs have revealed diverse strategies to downregulate antigen presentation, increase the expression of additional coinhibitory molecules, and create an immunosuppressive TME (56). While ICI therapy depends on reactivating exhausted T cells, many cancers evade T cell recognition by downregulating MHC-I and depleting tumor neoantigens, thereby limiting antigen presentation (17, 22, 56). Indeed, nonresponders to ICI therapy in head and neck cancers have low intratumoral CD8^+^ T cell infiltration and frequently exhibit low expression of MHC-I molecules on tumor cells (14–16). The MHC-I levels on HPV+ HNC cells are consistently lower than those in normal tissue (21, 23, 24). The downregulation of MHC-I is likely a key mechanism underlying the poor response rate in HPV+ HNC patients to ICI therapy despite high PD-L1 expression (1, 11, 12). Thus, restoring MHC-I could be a promising strategy to enhance tumor antigen presentation and antitumor CD8^+^ T cell activity.

## Materials and Methods

For detailed materials and methods, see *SI Appendix*.

HPV-positive and HPV-negative head and neck cancer cell lines, along with normal keratinocytes and murine mEERL cells, were cultured under defined conditions and used for in vitro and in vivo studies. Syngeneic C57BL/6J mouse tumor models were employed to assess tumor growth, survival, immune modulation, CD8^+^ T cell depletion, and responses to anti–PD-1 therapy. A TurboID proximity-labeling screen combined with mass spectrometry identified MARCHF8-interacting proteins, which were validated by immunoprecipitation, western blotting, and mutant analyses. Immune phenotyping was performed using flow cytometry and single-cell RNA sequencing, while gene expression and T cell function were evaluated by RT-qPCR, ELISA, and cytotoxicity assays. Statistical analyses included Seurat-based scRNA-seq workflows, *t* tests, and Kaplan–Meier survival analyses.

## Supplementary Material

Appendix 01 (PDF)

Dataset S01 (XLSX)

Dataset S02 (XLSX)

Dataset S03 (XLSX)

## Data Availability

scRNA-seq data have been deposited in NCBI GEO (GSE294715) (41). All other data are included in the manuscript and/or supporting information.
