# Supplementary material for: The membrane-associated ubiquitin ligase MARCHF8 degrades MHC-I in HPV-positive head and neck cancer for immune evasion
Source: Proc Natl Acad Sci U S A. 2026 Mar 9;123(11):e2525730123. doi: 10.1073/pnas.2525730123 (PMC12994185; doi:10.1073/pnas.2525730123)
Supplement: Supplementary file 1 — Appendix 01 (PDF) [file pnas.2525730123.sapp.pdf]

MATERIALS AND METHODS

Cell culture. HPV+ HNC (SCC2, SCC90, and SCC152) and HPV- HNC (SCC1, SCC9, and SCC19) cells were purchased from the American Type Culture Collection (ATCC) (Manassas, VA), and 293FT cells were purchased from Thermo Fisher (Waltham, MA). These cells were cultured in Dulbecco's modified Eagle's medium (DMEM) supplemented with 10% fetal bovine serum (FBS) and penicillin/streptomycin (Thermo Fisher) as described[1–4]. The N/Tert-1 cell lines were obtained from Dr. Iain Morgan and maintained in keratinocyte serum-free medium supplemented with epidermal growth factor (EGF), bovine pituitary extract, and penicillin/streptomycin as previously described [5–7]. The mouse oropharyngeal epithelial (MOE) cell line mEERL was cultured in E-medium (DMEM and F12 media supplemented with 0.005% hydrocortisone, 0.05% transferrin, 0.05% insulin, 0.0014% triiodothyronine, 0.005% EGF, and 2% FBS) as previously described [8,9].

Mouse experiments. C57BL/6J mice were purchased from Jackson Laboratory (Bar Harbor, ME) and maintained following the USDA guidelines. Six to eight-week-old mice were injected with 5×10^5 mEERL cells subcutaneously into the rear right flank (n = 10 per group). Tumor volume was measured twice weekly and calculated using the equation: volume = (width^2 X length)/2. Animals were euthanized when tumor volume reached 2,000 mm^3, as previously described in [10]. Conversely, mice were considered tumor-free when no measurable tumor was detected for 12 weeks. Survival curves were generated by Kaplan–Meier analysis, standardizing for a tumor volume of 1,000 mm^3. The Michigan State University Institutional Animal Care and Use Committee (IACUC) approved experiments involving live animals in accordance with National Institutes of Health guidelines (Approval Number PROTO202400214). CD8$^+$ T cells in C57BL/6J mice were depleted by intraperitoneal injection of 100 µg of an anti-CD8α neutralizing antibody (BioXcell, clone 2.43) every three days for 30 days, starting two days before tumor cell injection. An IgG2a isotype control antibody was used. For anti-PD-1 treatment, C57BL/6J mice were intraperitoneally injected with 200 µg of an anti-PD-1 antibody (BioXcell, clone RMP1-14) every three days for four times starting from 24 days post-injection when the average tumor size in the mice injected with mEERL/scr cells reached 200 mm^3. An IgG2b isotype control antibody was used.

TurboID screen

All TurboID plasmids were constructed using the In-Fusion Cloning system (Takara Bio). The HA-tagged TurboID gene (3xHA-TurboID) was amplified from 3xHA-TurboID-NLS pcDNA3 (Addgene, #107171) and inserted into the empty pCW57.1 vector (Addgene, #41393) using the NheI and BamHI restriction enzyme (RE) sites, with an AgeI RE site added to the 3' primer. pCW57.1-3xHA-TurboID was used as the control plasmid. The human MARCHF8 gene (Horizon Discovery, MHS6278-202807570) was amplified by PCR and inserted into pCW57.1-3xHA-TurboID at the AgeI and BamHI RE sites. SCC152 and N/Tert-1/E6E7 cell lines stably expressing 3xHA-TurboID-MARCHF8 or 3xHA-TurboID alone as control were generated by lentiviral transduction and puromycin selection. 293T cells (ATCC, CRL-3216) were transfected with each construct and third-generation lentiviral packaging plasmids (Cell BioLabs, VPK-206) using Lipofectamine 3000 (Thermo Fisher) according to the manufacturer's recommendations. The transfected cells were incubated at 37°C for 6 hours, replenished with fresh medium, and incubated at 37°C for 72 hours. The culture media were filtered through a 0.45-µm filter and added to the cells with polybrene (4 µg/ml; Santa Cruz Biotechnology, Dallas, TX). At 72 hours after transduction, stable cell lines were selected with puromycin (0.5 g/ml; Thermo Fisher) and verified for TurboID-MARCHF8 fusion protein or TurboID expression, and for their localization, using immunofluorescence and western blotting. For immunofluorescence, cells grown on glass coverslips were fixed in 3% (wt/vol) paraformaldehyde/phosphate-buffered saline (PBS) for 10 min and permeabilized with 0.4% (wt/vol) Triton X-100/PBS for 15 min. For labeling fusion proteins, a mouse anti-hemagglutinin (HA) antibody (1:1000; 12CA5; Covance) was used. The primary antibody was detected using Alexa Fluor 488-conjugated or 568–conjugated goat anti-mouse (1:1000; A11001 or A11004; Thermo Fisher Scientific). Alexa Fluor 488–conjugated or 568-conjugated streptavidin (1:1000; S32354 or S11226; Thermo Fisher Scientific) was used to detect biotinylated proteins. DNA was detected with Hoechst dye 33342. Coverslips were mounted with 10% (wt/vol) Mowiol 4-88 (Polysciences). Epifluorescence images were captured using a Nikon Eclipse NiE (20×/0.75 Plan Apo Nikon objective) microscope. To analyze total

cell lysates by western blot, 1.2 × 106 cells were lysed in SDS–PAGE sample buffer, boiled for 5 min, and sonicated to shear DNA. Proteins were separated on 4–20% gradient gels (Mini-PROTEAN TGX; Bio-Rad, Hercules, CA) and transferred to a nitrocellulose membrane (Bio-Rad). After blocking with 10% (vol/vol) adult bovine serum and 0.2% Triton X-100 in PBS for 30 min, the membrane was incubated with a rabbit anti-HA antibody (1:20000; ab9110; Abcam) overnight, washed with PBS, and detected using horseradish peroxidase (HRP)–conjugated anti-rabbit (1:40,000; G21234; Invitrogen). Antibody signals were detected using enhanced chemiluminescence via a Bio-Rad ChemiDoc MP System (Bio-Rad, Hercules, CA). Following HA detection, the membrane was quenched with 30% H2O2 for 30 min. To detect biotinylated proteins, the membrane was incubated with HRP-conjugated streptavidin (1:40,000; ab7403; Abcam) in 0.4% Triton X-100 in PBS for 45 min. The stable cell lines were maintained in DMEM (Corning, 10-013-CV) supplemented with 10% FBS. Large-scale TurboID pulldowns were performed in triplicate as described in [11]. Briefly, four 10 cm dishes at 80% confluency were incubated with doxycycline (1 g/ml) for 18 hours, then biotinylated with 50 µM biotin for 4 hours. Cells were rinsed twice with PBS and lysed in a 50 mM Tris buffer, pH 7.4, containing 8 M urea, protease inhibitors (Thermo Fisher, 87785), and DTT, then incubated with universal nuclease (Thermo Fisher, 88700) and sonicated to further shear the DNA. Lysates were precleared with Gelatin Sepharose 4B beads (GE Healthcare, 17095601) for 2 hours and then incubated with Streptavidin Sepharose High-Performance beads (GE Healthcare, 17511301) for 4 hours. Streptavidin beads were washed four times with 50 mM Tris, pH 7.4, and 8 M urea, and resuspended in 50 mM ammonium bicarbonate with 1 mM biotin.

Mass spectrometry

Protein samples were reduced, alkylated, and digested using filter-aided sample preparation with porcine trypsin (Promega) [12]. Tryptic peptides were separated by reverse-phase XSelect CSH C18 2.5 m resin (Waters) on an in-line 150 x 0.075 mm column using an UltiMate 3000 RSLCnano system (Thermo Fisher). Peptides were eluted with a 60-min gradient from 98:2 to 65:35 buffer A:B (Buffer A = 0.1% formic acid, 0.5% acetonitrile; Buffer B = 0.1% formic acid, 99.9% acetonitrile). Eluted peptides were ionized by electrospray (2.4 kV) and analyzed by mass spectrometry (MS) on an Orbitrap Fusion Tribrid mass spectrometer (Thermo Fisher). MS data were acquired using the Fourier Transform Mass Spectrometer in profile mode at a resolution of 240,000 over the m/z range 375 to 1500. Following higher-energy collisional dissociation activation, MS/MS data were acquired using the ion trap analyzer in centroid mode and the normal mass range, with a normalized collision energy of 28-31%, depending on the charge state and precursor selection range. Proteins were identified by database search using MaxQuant (Max Planck Institute) for label-free quantification, with a parent ion tolerance of 2.5 ppm and a fragment ion tolerance of 0.5 Da. Scaffold Q+S (Proteome Software) was used to verify MS/MS-based peptide and protein identifications. Protein identifications were accepted if they could be established with less than 1.0% false discovery and contained at least two identified peptides. Protein probabilities were assigned by the Protein Prophet algorithm [13]. Protein interaction candidates were considered if identified in at least two replicate runs and enriched over the TurboID-only control.

Immunoprecipitation (IP) and western blotting

Whole-cell lysates from cultured cells were prepared in radioimmunoprecipitation assay (RIPA) buffer (Abcam, Waltham, MA) containing a protease inhibitor cocktail, according to the manufacturer's instructions (Roche, Mannheim, Germany). In the IFN-γ treatment experiment, cells were incubated with 1 ng/ml of human IFN-γ for 16 hours. Whole-cell lysates from paraffin-embedded tumor tissues were prepared using the Qproteome FFPE Tissue Kit (Qiagen, Germantown, MD). Total protein concentrations were determined using the Pierce BCA Protein Assay Kit (Thermo Fisher). IP was performed using the Pierce Classic Magnetic IP/Co-IP Kit (Thermo Fisher). Briefly, 25 l of protein A/G magnetic beads were incubated with 5 g of specific antibodies (**Table S1**) for 2 hours. 1 mg of whole-cell lysate was incubated with antibody-coupled beads at 4 °C overnight. Western blotting was performed with 10-20 µg of total protein using antibodies listed in **Table S1**, as previously described [14]. Band densities were determined using NIH ImageJ software and normalized to the β-actin band intensity.

Mutant MARCHF8 expression

The 3XHA-hMARCHF8-W114A coding sequence was amplified from pCAGGS-3xHA-MARCHF8-W114A (provided by Dr. Yong-Hui Zheng) and the HA-tagged wild-type MARCHF8 coding sequence was generated from pCAGGS-3XHA-hMARCHF8 (also provided by Dr. Yong-Hui Zheng) using the primers listed in **Table S2**. It was cloned into the SpeI and AgeI restriction sites of the pLenti6/V5-D-TOPO vector (Addgene, #22945). The DIRT domain was deleted from pLenti6-3xHA-MARCHF8 using the QuikChange II XL Site-Directed Mutagenesis Kit (Agilent, 200522) and primers from **Table S2**. shRNAs targeting human MARCHF8 were purchased from Sigma-Aldrich. The sgRNAs targeting mouse Marchf8 were designed with the web-based software ChopChop 5 [15]. These sgRNAs were synthesized and cloned into the lentiCRISPR v2-blast plasmid (Addgene, #83480) using ligating duplex oligonucleotides with BsmBI RE sites (IDT). All shRNA and sgRNA sequences are listed in **Tables S3 and S4**, respectively. Lentiviruses containing shRNA or sgRNA were produced in 293FT cells with packaging constructs pCMV-VSV-G (Addgene, #8454) and pCMV-Delta R8.2 (Addgene, #12263). The viruses were collected 48 hours after transfection and concentrated by ultracentrifugation at 25,000 rpm for 2 hours. Cells were incubated with the lentiviruses for 48 hours in the presence of 8 μg/ml polybrene and selected with 8 μg/ml blasticidin.

Flow cytometry

Single cells were isolated from tumor tissue, counted, and analyzed using antibodies listed in **Table S1**, as previously described. A cell viability assay was performed by staining cells with the Zombie NIR fixable viability dye (BioLegend). Cells for IFN, Gzma, Perforin, and Ki-67 intracellular staining were collected from the tumor tissues of mice injected with 200 μg of Brefeldin A (BioLegend) 6 hours before euthanasia. The cells were surface-stained with CD45, CD3, CD4, CD8, and NK1.1 antibodies listed in **Table S1** after viability staining with Zombie NIR. Intracellular IFN, Gzma, Perforin, and Ki-67 staining was performed using the FoxP3 staining kit (eBiosciences) (San Diego, CA) according to the manufacturer's instructions. Cells for immune cell profiling were stained with antibodies using BD Brilliant staining buffer and analyzed on either a five-laser Cytek Aurora spectral cytometer or a three-laser LSRII flow cytometer (BD Biosciences) in the MSU Flow Cytometry Core Facility. High-dimensional reduction and clustering analyses of flow cytometric data were performed in FCS Express (v7) using the following pipeline to evaluate global TIL changes in response to treatments for each experimental day. First, the data were scaled automatically, then normalized by standard deviation, centered by subtracting the mean, and rescaled to a minimum of 0 and a maximum of 1. This ensured a unimodal distribution of the data and resolved dim populations. The data were then gated manually to remove debris, doublets, dead cells, and CD45-negative populations. Next, a UMAP analysis was performed with the following settings to visualize distinct sub-populations: all files used, all phenotype parameters used, except for CD45 and Zombie NIR in the FSC files, Neighbors = 50, Minimum Low Dim Distance = 0.9 or 1, Iterations = 500, Random Seed = 886341810. After the UMAP analysis, FlowSOM was used to cluster the data. FlowSOM settings are as follows: all files used, New Scaling = unchecked, Cluster Centroids Initialization Method = Random Cells, Training Decay Function = linear, 2D Grid Neighborhood Function = Boxcar, 2D Grid Distance Metric = Chebyshev, Coarse Training Options = 20, Fine-Tune training Options = 10, Automatic Neighborhood Spread = Checked, Minimum Spanning Tree = Batch SOM Cluster Assignments, Number of Clusters = 18, Sampling Fraction = 1, Number of Samplings = 100, Clustering Algorithms = Hierarchical, Random Seed = 898787008. Next, clusters were assigned a designation based on their phenotype. UMAP scatterplots are shown with color-coded FlowSOM clusters. Clusters were manually annotated based on expression patterns of cellular markers, and dotted lines were drawn to highlight clusters with similar immunophenotypes. Manual gating was performed in FlowJo (v10) to quantify the percentages of live-cell and immune-cell populations.

Quantitative reverse transcription-PCR (RT-qPCR)

Total RNA was isolated using the RNeasy Plus Mini Kit (Qiagen, Germantown, MD). First-strand cDNA was synthesized from 2 g of total RNA using reverse transcriptase (Roche, Mannheim, Germany). Quantitative PCR (qPCR) was performed in a 20 l reaction mixture containing 10 l of SYBR Green Master Mix (Applied Biosystems), 5 l of 1 μM primers, and 100 ng of cDNA template, using a Bio-Rad CFT

Connect thermocycler. Data were normalized to glyceraldehyde 3-phosphate dehydrogenase (GAPDH) or β-actin. Primers used in qPCR (**Table S2**) were synthesized by IDT.

Intratumoral immune cell isolation

Immune cells were isolated from tumor tissues as previously described [16]. Tumors were surgically removed from mice, minced in RPMI medium, and incubated with 0.5 mg/ml collagenase IV (Millipore-Sigma) and 1,000 IU/ml DNase I (Millipore-Sigma) at 37 °C for 1 hour, with vortexing every 15 min. After digestion, cells were resuspended in RPMI supplemented with 10% FBS and filtered through a 40 M cell strainer. Red blood cells (RBCs) were lysed by adding 1.5 ml of RBC lysis buffer (Millipore-Sigma) for less than 5 min.

Single-cell RNA sequencing (scRNA-seq)

ScRNA-seq libraries were generated using the 10X Genomics Chromium Single Cell 3` Reagent Kit (v2 Chemistry) and the Chromium Single Cell Controller, as described in the previously published study [17]. Briefly, FACS-sorted cells were loaded into each reaction for gel bead-in-emulsion (GEM) generation and cell barcoding. Reverse transcription of the GEM (GEM-RT) was performed using a Veriti 96-Well Fast Thermal Cycler (Applied Biosystems) at 53 ^{o}C for 45 min, 85 ^{o}C for 5 min, and a 4 °C hold. cDNA amplification was performed after GEM-RT cleanup with Dynabeads MyOne Silane (ThermoFisher Scientific) using the same thermocycler (98 ^{o}C for 3 min, 98 ^{o}C for 15 seconds, 67 ^{o}C for 20 seconds, 72 ^{o}C for 1 min, for 12 cycle repeats followed by a 72 ^{o}C for 1 min and a 4 ^{o}C hold). The amplified cDNA was cleaned up with SPRIselect Reagent Kit (Beckman Coulter, Brea, CA), followed by library construction, including fragmentation, end repair, adaptor ligation, and library amplification. An Agilent 2100 Bioanalyzer (Agilent, Santa Clara, CA) was used for library quality control. Libraries were sequenced on an Illumina HiSeq4000 using a paired-end flow cell: Read 1, 26 cycles; i7 index, 8 cycles; Read 2, 98 cycles.

T cell isolation and stimulation

Mouse spleens were mechanically disrupted through a 100-m filter and subjected to magnetic bead negative enrichment for CD8+ T cells using a CD8+ T-cell Isolation Kit (STEMCELL Technologies). Isolated CD8+ T cells were cultured in RPMI (Gibco) containing 10% FBS, L-glutamine, penicillin/streptomycin, and 50 M 2-mercaptoethanol. Isolated CD8+ T cells (1 × 106 cells/ml) were stimulated with Dynabeads Mouse T-Activator CD3/CD28 (Invitrogen). Stimulated cells were incubated in media supplemented with 10 ng/mL of recombinant mouse IL-2 (rmIL-2) (ThermoFisher).

Enzyme-linked immunosorbent assay (ELISA)

Isolated CD8+ T-cells were co-cultured with mitomycin-C-treated mEERL cells expressing scramble or Marchf8 sgRNA in RPMI containing 10 ng/mL rmIL-2 and Dynabeads Mouse T-Activator CD3/CD28 (Invitrogen) for T-cell Expansion and Activation kit for 3 days. Cell culture supernatants were collected and used to quantify IFN-γ production by ELISA (Biolegend, #430801) according to the manufacturer's instructions.

Lactate dehydrogenase release assay

To measure CD8+ T-cell cytotoxicity, one million isolated CD8+ T cells (effector) were incubated with 10,000 mEERL cells (target) transduced with scramble or Marchf8 sgRNA for 24 hours. Cell culture supernatant was collected, and cytotoxicity was determined by measuring lactate dehydrogenase (LDH) concentrations in the supernatant using the Cytotoxicity Detection Kit (Millipore Sigma). As controls, spontaneous and maximum cell lysis were assessed in the absence of CD8+ T cells and by treating the mEERL cells with 1% SDS, respectively.

Quantification and statistical analysis

As described in our previous study [18], reads from scRNA-seq libraries were demultiplexed, aligned to the mm10 mouse reference, barcode processed, and Unique Molecular Identifier (UMI) counts obtained using the 10X Genomics Cell Ranger (v2.0.1) pipeline. Datasets were subsequently analyzed using the R Seurat package. Principal Component Analysis (PCA) was performed on the combined samples. Cells

with feature counts between 2,500 and 200 and/or high mitochondrial content (>5%) were filtered out. The global-scaling normalization method "LogNormalize" in Seurat was used to normalize gene expression. Highly variable genes in each dataset were identified, and the intersecting top 2,000 genes across datasets were used for clustering and downstream analyses. The first 30 principal components (PCs) were used to visualize the resulting clusters using the Uniform manifold approximation and projection (UMAP) method. The FindAllMarkers function in Seurat was used to identify DEGs between clusters, with a Bonferroni-adjusted P value <0.05 as the significance threshold. Other data were analyzed using GraphPad Prism (San Diego, CA) and presented as mean ± standard deviation. Statistical significance was determined using an unpaired Student's t-test. P values <0.05 are considered statistically significant. Distributions of time-to-event outcomes (e.g., survival time) were summarized using Kaplan–Meier curves and compared across groups using the log-rank test, with p = 0.01.

REFERENCES

1. Westrich, J. A. *et al.* Human Papillomavirus 16 E7 Stabilizes APOBEC3A Protein by Inhibiting Cullin 2-Dependent Protein Degradation. *J Virol* 92, (2018).
2. Ludwig, S. *et al.* Proteomes of exosomes from HPV(+) or HPV(-) head and neck cancer cells: differential enrichment in immunoregulatory proteins. *Oncoimmunology* 8, 1593808 (2019).
3. Ayuso, J. M. *et al.* Effects of culture method on response to EGFR therapy in head and neck squamous cell carcinoma cells. *Sci Rep* 9, 12480 (2019).
4. Ou, D. *et al.* miR-340-5p affects oral squamous cell carcinoma (OSCC) cells proliferation and invasion by targeting endoplasmic reticulum stress proteins. *Eur J Pharmacol* 920, 174820 (2022).
5. Kume, T., Deng, K. & Hogan, B. L. Minimal phenotype of mice homozygous for a null mutation in the forkhead/winged helix gene, Mf2. *Mol Cell Biol* 20, 1419–25 (2000).
6. Evans, M. R. *et al.* Human Papillomavirus 16 E2 Regulates Keratinocyte Gene Expression Relevant to Cancer and the Viral Life Cycle. *J Virol* 93, (2019).
7. James, C. D. *et al.* SAMHD1 Regulates Human Papillomavirus 16-Induced Cell Proliferation and Viral Replication during Differentiation of Keratinocytes. *mSphere* 4, (2019).
8. Spanos, W. C. *et al.* Immune response during therapy with cisplatin or radiation for human papillomavirus-related head and neck cancer. *Arch Otolaryngol Head Neck Surg* 135, 1137–46 (2009).
9. Allen-Hoffmann, B. L. *et al.* Normal growth and differentiation in a spontaneously immortalized near-diploid human keratinocyte cell line, NIKS. *J Invest Dermatol* 114, 444–55 (2000).
10. Cicchini, L. *et al.* Suppression of Antitumor Immune Responses by Human Papillomavirus through Epigenetic Downregulation of CXCL14. *mBio* 7, (2016).
11. May, D. G. & Roux, K. J. BioID: A Method to Generate a History of Protein Associations. *Methods Mol Biol* 2008, 83–95 (2019).
12. Wiśniewski, J. R., Zougman, A., Nagaraj, N. & Mann, M. Universal sample preparation method for proteome analysis. *Nat Methods* 6, 359–62 (2009).
13. Nesvizhskii, A. I., Keller, A., Kolker, E. & Aebersold, R. A statistical model for identifying proteins by tandem mass spectrometry. *Anal Chem* 75, 4646–58 (2003).
14. Warren, C. J. *et al.* APOBEC3A functions as a restriction factor of human papillomavirus. *J Virol* 89, 688–702 (2015).
15. Labun, K. *et al.* CHOPCHOP v3: expanding the CRISPR web toolbox beyond genome editing. *Nucleic Acids Res* 47, W171–W174 (2019).
16. O'Connell, P. *et al.* Adenoviral delivery of an immunomodulatory protein to the tumor microenvironment controls tumor growth. *Mol Ther Oncolytics* 24, 180–193 (2022).
17. Wang, J. *et al.* Integrative scATAC-seq and scRNA-seq analyses map thymic iNKT cell development and identify Cbfβ for its commitment. *Cell Discov* 9, 61 (2023).
18. Wang, J. *et al.* Single-cell analysis reveals differences among iNKT cells colonizing peripheral organs and identifies Klf2 as a key gene for iNKT emigration. *Cell Discov* 8, 75 (2022).

Table S1. List of the antibodies

Antibody	Specificity	Source	Catalog	RRID	Experiment
HPV16 E7 (clone ED17)		Santa Cruz	SC-6981	AB_627745	Western blot
MARCHF8	Human/Mouse	ThermoFisher	PA5-88893	AB_2805201	Western blot
MARCHF8	Human/Mouse	Proteintech	14119-1-AP	AB_2140168	IP
HLA class I A/B/C	Human	Proteintech	15240-1-AP	AB_1557426	IP-WB
ubiquitin	Human/Mouse	Proteintech	10201-2-AP	AB_671515	IP
FITC-HLA-A/B/C	Human	BioLegend	311404	AB_314872	Flow cytometry
PE-H-2Ld/H-2Db	Mouse	BioLegend	114507	AB_313588	Flow cytometry
Brilliant Violet 510-Ly6C	Mouse	BioLegend	128033	AB_2562351	Flow cytometry
PerCP-Ly6G	Mouse	BioLegend	127653	AB_2616999	Flow cytometry
PE/Dazzle 594 I-A/I-E	Mouse	BioLegend	107647	AB_2565979	Flow cytometry
PE-Granzyme A	Mouse	BioLegend	149704	AB_2565310	Flow cytometry
Brilliant Violet 650-Ki-67	Human/Mouse	BioLegend	151215	AB_2876504	Flow cytometry
Brilliant Violet 421-Perforin	Mouse	BioLegend	154319	AB_3083137	Flow cytometry
APC-IFNγ	Mouse	BioLegend	163513	AB_3097457	Flow cytometry
APC/Fire 750-CD8a	Mouse	BioLegend	100765	AB_2572113	Flow cytometry
Brilliant Violet 605-F4/80	Mouse	BioLegend	123133	AB_2562305	Flow cytometry
Brilliant Ultraviolet 395-CD45	Mouse	Thermo Fisher	363-0451-80	AB_2925263	Flow cytometry
Super Bright 780-CD11b	Mouse	ThermoFisher	78-0112-80	AB_2722925	Flow cytometry
APC/Fire 810-CD3	Mouse	BioLegend	100268	AB_2876392	Flow cytometry
PE/Cy7-NK1.1	Mouse	BioLegend	108714	AB_389364	Flow cytometry
PE-CD11c	Mouse	BioLegend	117308	AB_313777	Flow cytometry
FITC-CD4	Mouse	BioLegend	100510	AB_312713	Flow cytometry
Spark Blue 550-CD19	Mouse	BioLegend	115565	AB_2819827	Flow cytometry
PE/Fire 810-Tim3	Mouse	BioLegend	119745	AB_2922462	Flow cytometry
APC-LAG3	Mouse	BioLegend	125209	AB_10639935	Flow cytometry
PerCP-eFluor 710-PD-1	Mouse	ThermoFisher	46-9981-80	AB_11149347	Flow cytometry
Anti-mouse CD8α	Mouse	Bio X Cell	BE0061	AB_1125541	CD8 depletion
Anti-mouse PD-1	Mouse	Bio X Cell	BE0146	AB_10949053	PD-1 blocking
Rat IgG2a isotype control	Mouse	Bio X Cell	BE0089	AB_1107769	In vivo control
Rat IgG2b isotype control	Mouse	Bio X Cell	BE0090	AB_1107780	In vivo control

RRID, Research Resource Identifiers

Table S2. List of oligonucleotides

Name	Sequence	Experiment
Human GAPDH FWD	5`-GGAGCGAGATCCCTCCAAAAT-3`	RT-qPCR
Human GAPDH Rev	5`-GGCTGTTGTCATACTTCTCATGG-3`	RT-qPCR
Human HLA-A FWD	5`-CTTGTAAAGTGTGAGACAGC-3`	RT-qPCR
Human HLA-A Rev	5`-CTTCAAGTCACAAAGGGAAG-3`	RT-qPCR
Human HLA-B FWD	5`-ATGTGTAGGAGGAAGAGTTC-3`	RT-qPCR
Human HLA-B Rev	5`-GAAGAAATCCTGCATCTCAG-3`	RT-qPCR
Human HLA-C FWD	5`-CATCACTTGTAAAGCCTGAG-3`	RT-qPCR
Human HLA-C Rev	5`-CTCTTGAAGTCACAAAGGAG-3`	RT-qPCR
3X HA tag FWD	5`-TCCGGAACTAGTATGATCTTTTACCCATAC-3`	Cloning
Human MARCHF8 Rev	5`-TTACTAACCGGTTCAGACGTGAATGAT-3`	Cloning
Human MARCHF8 ∆DIRT FWD	5`-CATGGAGACCAAGCTGAAGCCAAGGAAGATCATGTGCT CAGTGAC-3`	Cloning
Human MARCHF8 ∆DIRT Rev	5`-GTCACTGAGCACATGATCTTCCTTGGCTTCAGCTTGGTC TCCATG-3`	Cloning

FWD, forward primer; Rev, reverse primer

Table S3. List of shRNAs

Name	Sigma-Aldrich TRC Clone ID	Sequence
Human MARCHF8 shRNA1	TRCN0000073233	5`-CTTGAGCTGAATGAGAGAATA-3`
Human MARCHF8 shRNA2	TRCN0000073234	5`-CCACTAACAGAGCCCAACTTT-3`
Human MARCHF8 shRNA3	TRCN0000073235	5`-CAGTGTAAAGTGTATGTGCAA-3`
Human MARCHF8 shRNA4	TRCN0000073236	5`-CTGGTCCTTGTATGTGCTCAT-3`
Human MARCHF8 shRNA5	TRCN0000073237	5`-CCTCCTTCTCTCGCACTTCTA-3`

Table S4. List of sgRNAs

Name	Sequence
Mouse MARCHF8 sgRNA1 FWD	5`-CACCG**AGGTGAGTATATGGGCCGTGAGG**-3`
Mouse MARCHF8 sgRNA1 Rev	5`-AAAC**CCTCACGGCCCATATACTCACCT**-3`
Mouse MARCHF8 sgRNA2 FWD	5`-CACCG**TATTAACGTCTGACCATGTGAGG**-3`
Mouse MARCHF8 sgRNA2 Rev	5`-AAAC**CCTCACATGGTCAGACGTTAATA**-3`
Mouse MARCHF8 sgRNA3 FWD	5`-CACCG**ACTACCAGCTTCGTCCAGAAAGG**-3`
Mouse MARCHF8 sgRNA3 Rev	5`-AAAC**CCTTTCTGGACGAAGCTGGTAGT**-3`

FWD, forward oligo; Rev, reverse oligo

Khalil et al., Fig. S1.

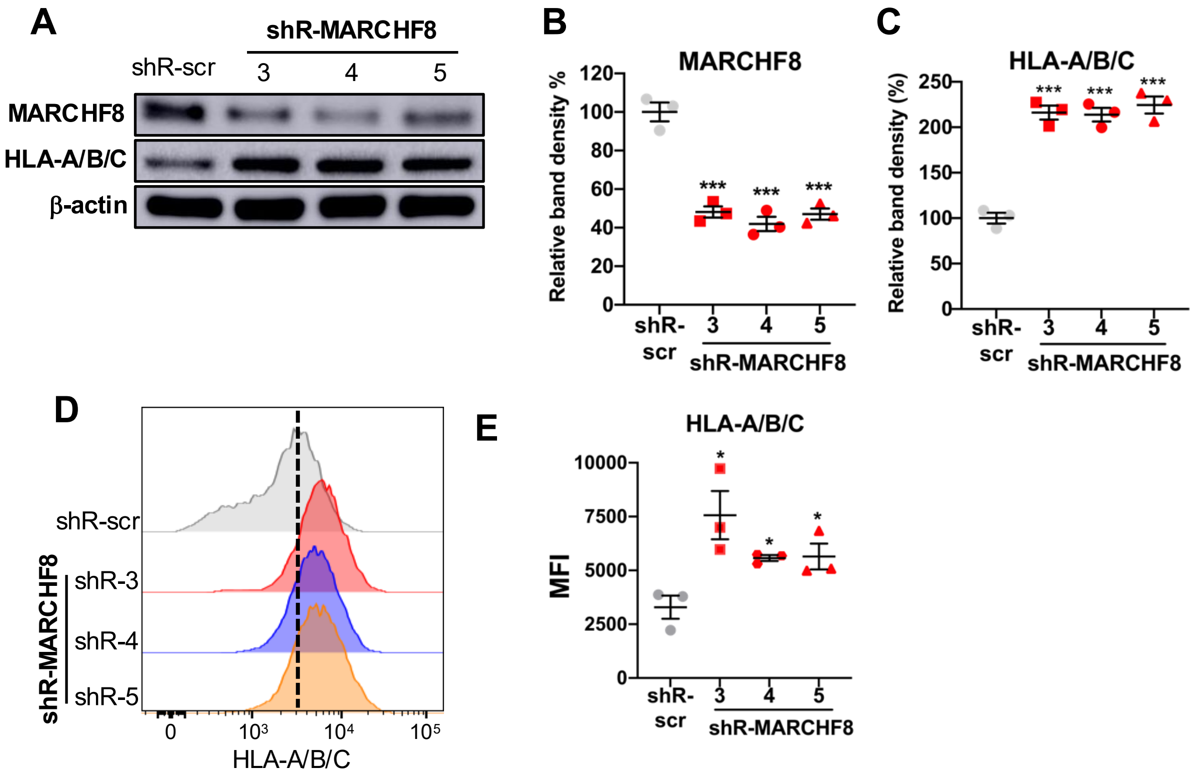

Fig. S1. MARCHF8 knockdown increases MHC-I protein levels in HPV+ HNC cells. HPV+ HNC cells (SCC2) were transduced with three lentiviral shRNAs against MARCHF8 (shR-MARCHF8) or scrambled shRNA (shR-scr). Protein levels of MARCHF8 and HLA-A/B/C were determined by western blotting (**A**-**C**). Relative band density was quantified using NIH ImageJ (**B** and **C**). Cell surface expression of HLA-A/B/C (**D** and **E**) proteins was analyzed by flow cytometry. MFI of three independent experiments is shown (**E**). The data shown are means ± SD of three independent experiments. *P* values were determined by Student's *t*-test. $*p < 0.05$, $***p < 0.001$.

Khalil et al., Fig.S2

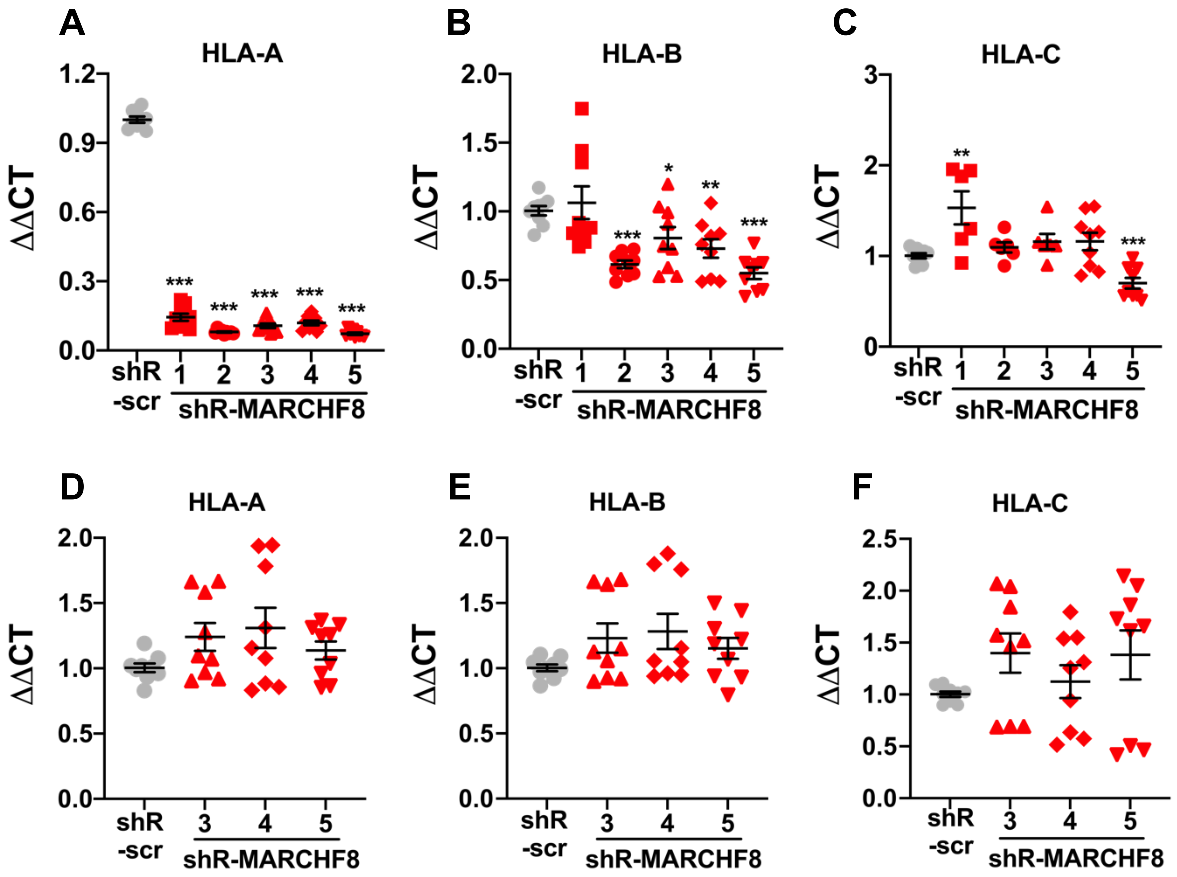

Fig. S2. mRNA expression levels of HLA-A, HLA-B, and HLA-C in HPV+ HNC cells with MARCHF8 knockdown. Two HPV+ HNC cell lines, SCC152 (**A-C**) and SCC2 (**D-F**), were transduced with five and three lentiviral shRNAs against MARCHF8 (shR-MARCHF8), respectively, or scrambled shRNA (shR-scr). The mRNA levels of HLA-A (**A** and **D**), HLA-B (**B** and **E**), and HLA-C (**C** and **F**) were assessed by RT-qPCR. The data shown are normalized by the GAPDH mRNA level as internal control. All experiments were repeated at least three times, and the data shown are means ± SD. *P* values were determined by Student's *t*-test. *$p < 0.05$, **$p < 0.01$, ***$p < 0.001$.

Khalil et al., Fig. S3

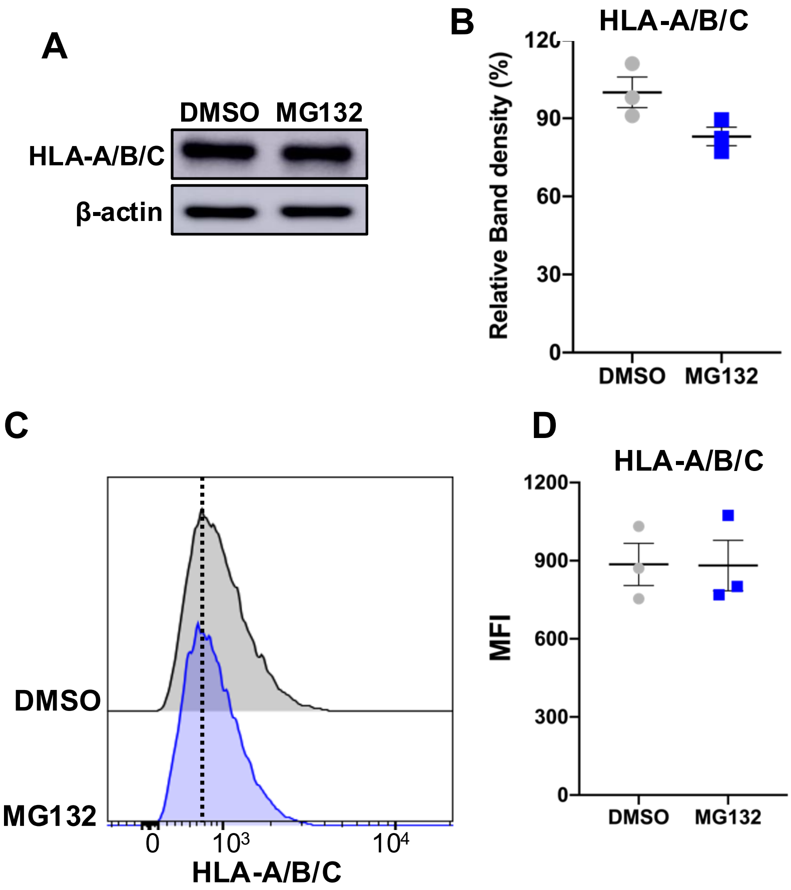

Fig. S3. The proteasome inhibitor MG132 did not increase the MHC-I protein level in HPV+ HNC cells. HPV+ HNC cells (SCC152) were treated with MG132 (proteasome inhibitor) for 16 hrs. Protein levels of HLA-A/B/C were determined by western blotting (**A**). Relative band density was quantified using NIH ImageJ (**B**). Cell surface expression of HLA-A/B/C (**C** and **D**) proteins was analyzed by flow cytometry. MFI of three independent experiments is shown (**D**). The data shown are means ± SD of three independent experiments. *P* values were determined by Student's *t*-test. No statistical significance was observed.

Khalil et al., Fig. S4.

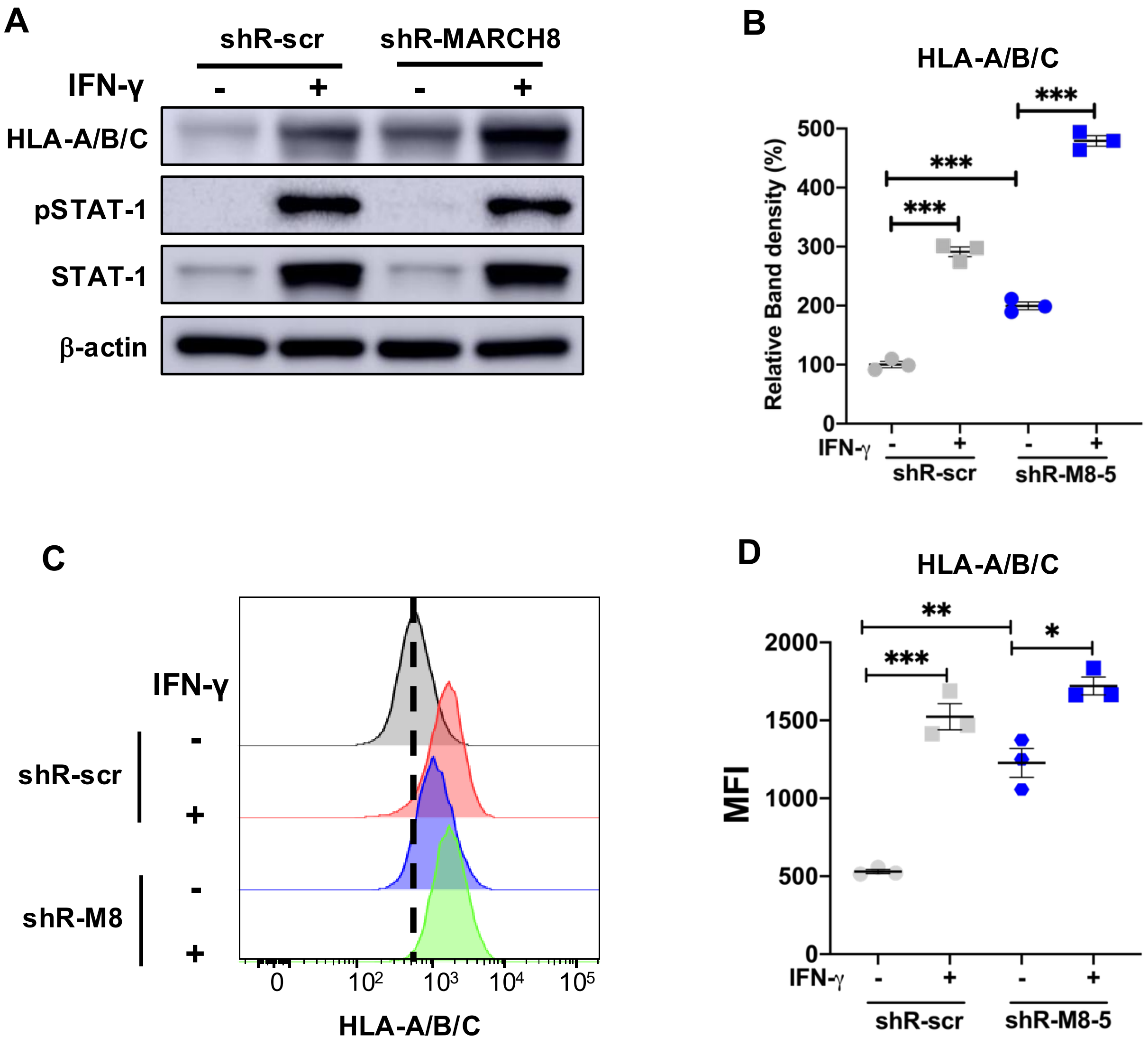

Fig. S4. Effect of IFNγ treatment on MHC-I expression in HPV+ HNC cells with MARCHF8 knockdown. SCC152 with shR-scr or shR-MARCHF8 (clone 5) were treated with 1 ng/ml of IFN-γ for 16 hours and whole cell lysates were prepared and analyzed by western blotting for using an anti-HLA-A/B/C antibody (**A**). STAT-1, pSTAT-1, and β-actin were used as controls. Relative band density was quantified using NIH ImageJ (**B**). Cell surface expression of HLA-A/B/C proteins was analyzed by flow cytometry (**C**). MFI of three independent experiments is shown (**D**). The data shown are means ± SD of three independent experiments. *P* values were determined by Student's *t*-test. $^{*}p < 0.05$, $^{***}p < 0.001$.

Khalil et al., Fig. S5.

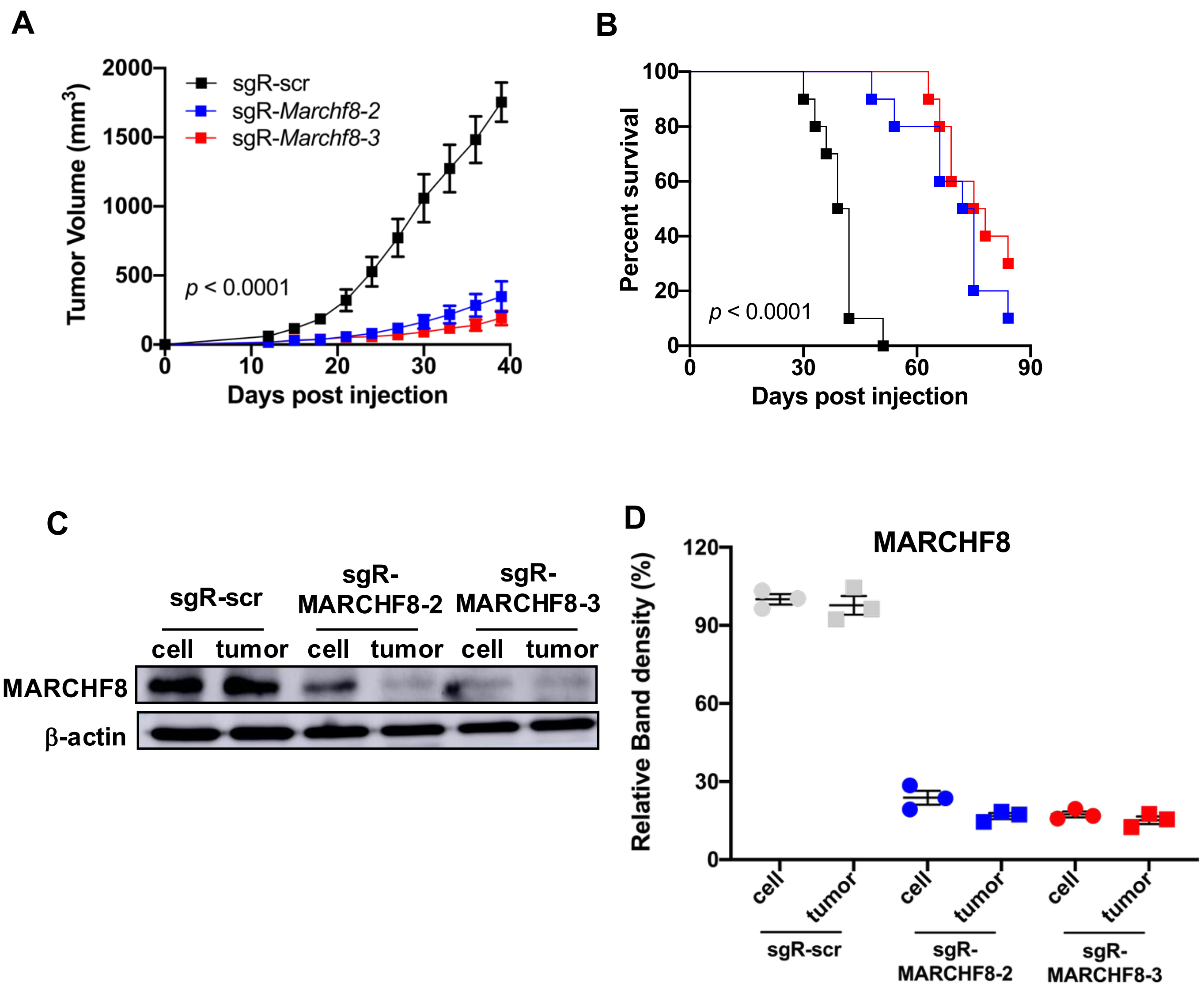

Fig. S5. *Marchf8* knockout in HPV+ HNC cells suppresses tumor growth in vivo. mEERL cells were transduced with lentiviral *Cas9* and one of two sgRNAs against *Marchf8* (sgR-*Marchf8-2* and sgR-*Marchf8-3*) or scrambled sgRNA (sgR-scr). mEERL/scr or mEERL/*Marchf8*$^{-/-}$ (sgR-*Marchf8-2* and sgR-*Marchf8-3*) cells were injected into the rear right flank of C57BL/6J mice (n = 10 per group). Tumor volume was measured twice a week (**A**). Survival rates of mice were analyzed using a Kaplan-Meier estimator (**B**). The time-to-event was determined for each group, with the event defined as a tumor size of 2,000 mm^3. The data shown are means ± SD. P values of mice injected with mEERL/*Marchf8*$^{-/-}$ cells compared with mice injected with mEERL/scr cells were determined for tumor growth (**A**) and survival (**B**) by two-way ANOVA analysis. Shown are representative of two independent experiments. MARCHF8 protein levels were determined in mEERL/scr or mEERL/*Marchf8*$^{-/-}$ (sgR-*Marchf8-2* and sgR-*Marchf8-3*) cells and tumor tissues by western blotting (**C**), and relative band density was quantified using NIH ImageJ (**D**). The data shown are means ± SD of three independent experiments.

Khalil et al., Fig.S6

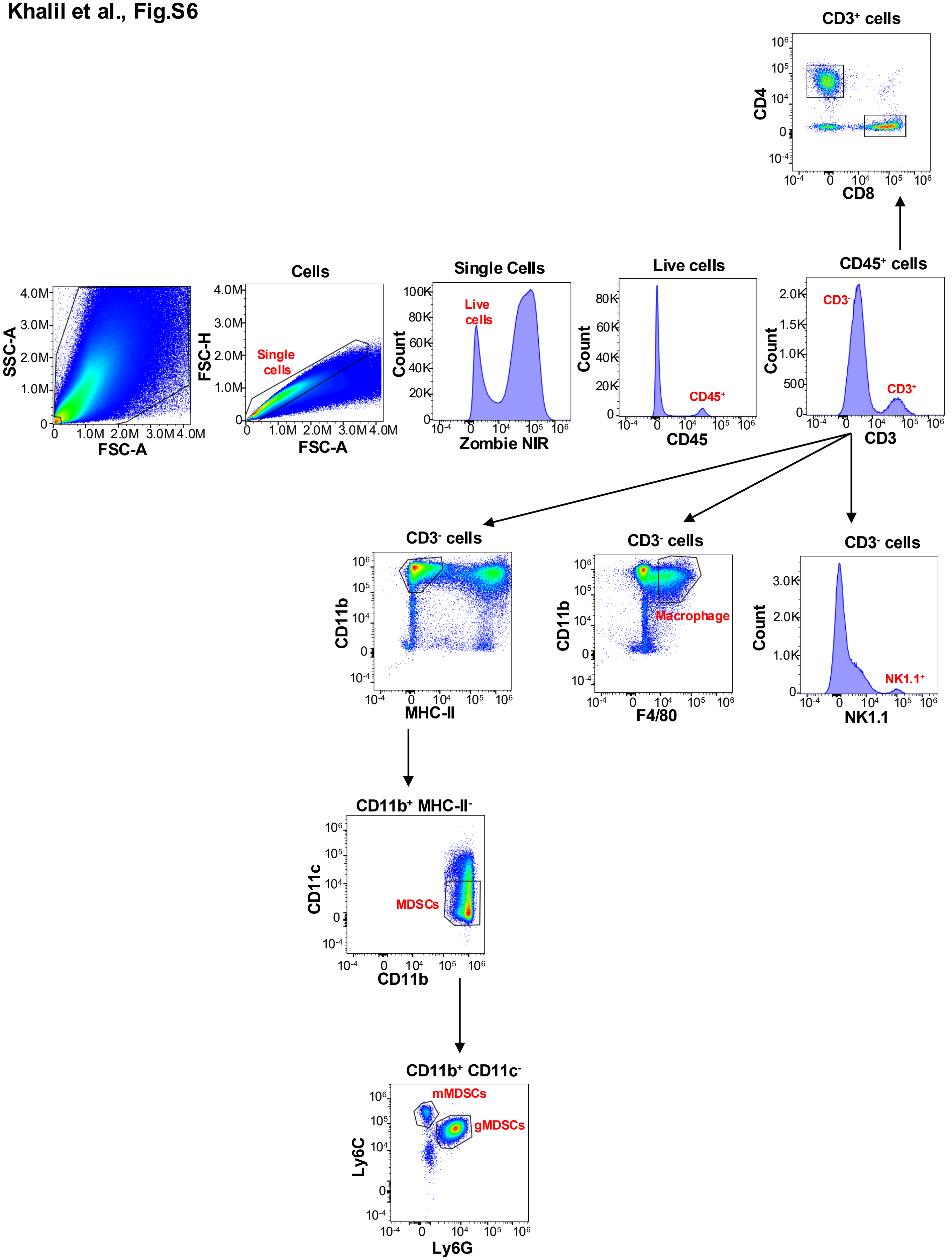

Fig. S6. Representative gating strategy for flow cytometry analysis. Single cells isolated from the tumor tissues were stained with an antibody cocktail containing antibodies for CD45, CD3, CD4, CD8, CD11b, CD11c, MHC-II, F4/80, NK1.1, Ly6C, and Ly6G and the viability dye Zombie NIR. The single cells were gated for live cells using Zombie NIR viability dye and subsequently gated for CD45 expression as a marker for immune cells. $CD45^{+}$ immune cells were classified into $CD3^{-}$ and $CD3^{+}$ cells based on CD3 expression. The $CD3^{+}$ T cells were branched into $CD4^{+}$ and $CD8^{+}$ T cells, while $CD3^{-}$ cells contain macrophages ($F4/80^{+}$), NK cells ($NK1.1^{+}$), and MDSCs ($CD11b^{+}$ $MHCII^{-}$). MDSCs were divided into granulocytic (gMDSC, $Ly6G^{+}$) and monocytic (mMDSC, $Ly6C^{+}$).

Khalil et al., Fig. S7

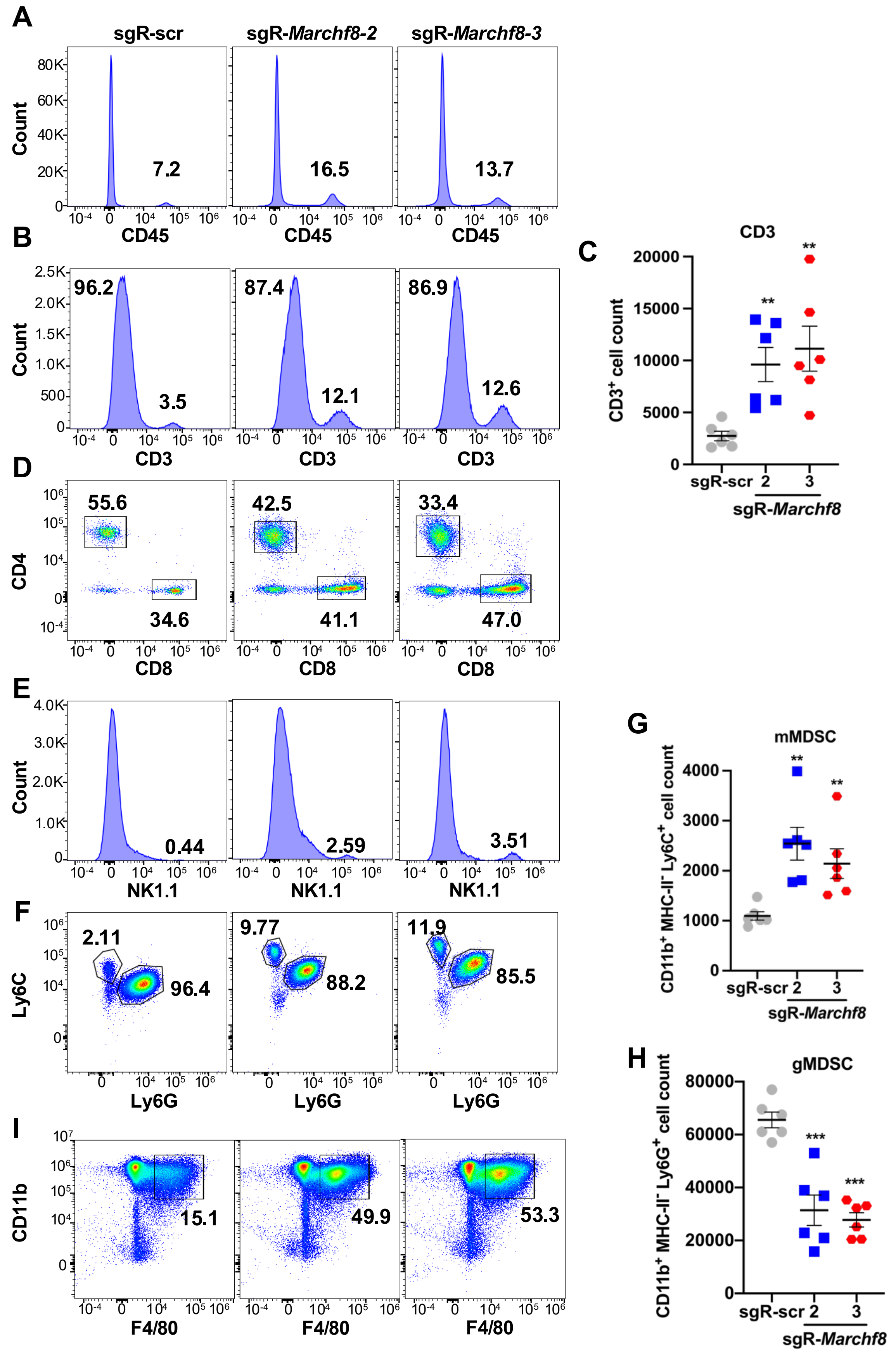

Fig. S7. ***Marchf8*** **knockout in HPV+ HNC cells increases NK cells and macrophages and decreases granulocytic MDSCs in the tumor tissues.** Tumors were isolated from C57BL/6J mice injected with mEERL/scr or mEERL/*Marchf8*$^{-/-}$ (sgR-Marchf8-2 and sgR-Marchf8-3) cells. Single cells isolated from the tumor tissues were stained with an antibody cocktail and analyzed by flow cytometry**.** Representative flow cytometry plots show CD45^{+} immune cells (**A**), CD3^{+} (**B** and **C**), CD4^{+} and CD8^{+} T cells (**D**), NK1.1^{+} NK cells (**E**), mMDSCs (CD11b^{+} MHC-II^{-} Ly6C^{+}) (F and I), and gMDSCs (CD11b^{+} MHC-II^{-} Ly6G^{+}) (**F-J**), and macrophages (CD11b^{+} F4/80^{+}) (**I**). MFI of three independent experiments is shown (**C, G,** and **H**). All experiments were repeated at least three times, and the data shown are means ± SD. *P* values were determined by Student's *t*-test. $^{**}p < 0.01$, $^{***}p < 0.001$.

Khalil et al., Fig.S8

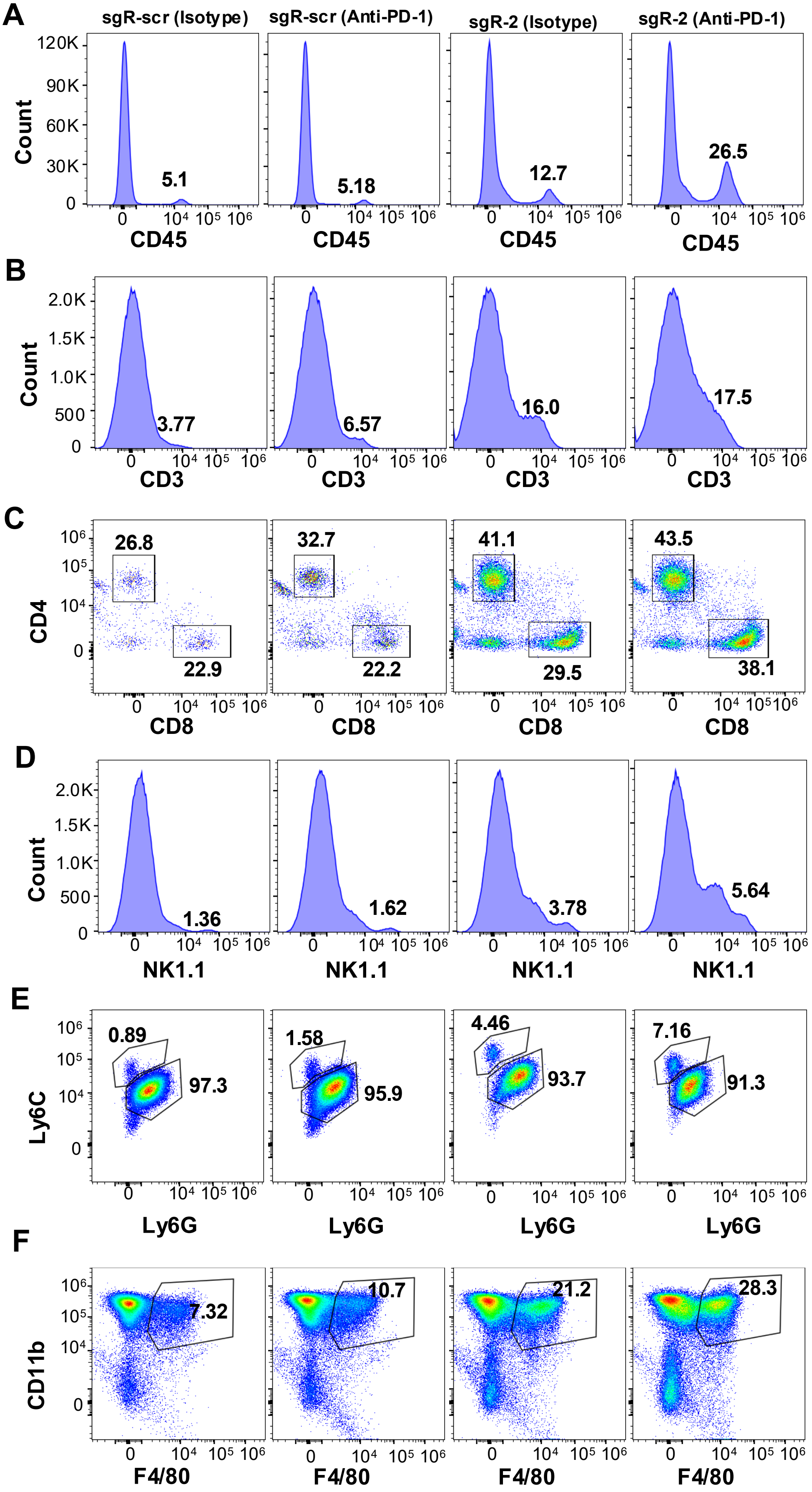

Khalil et al., Fig.S8

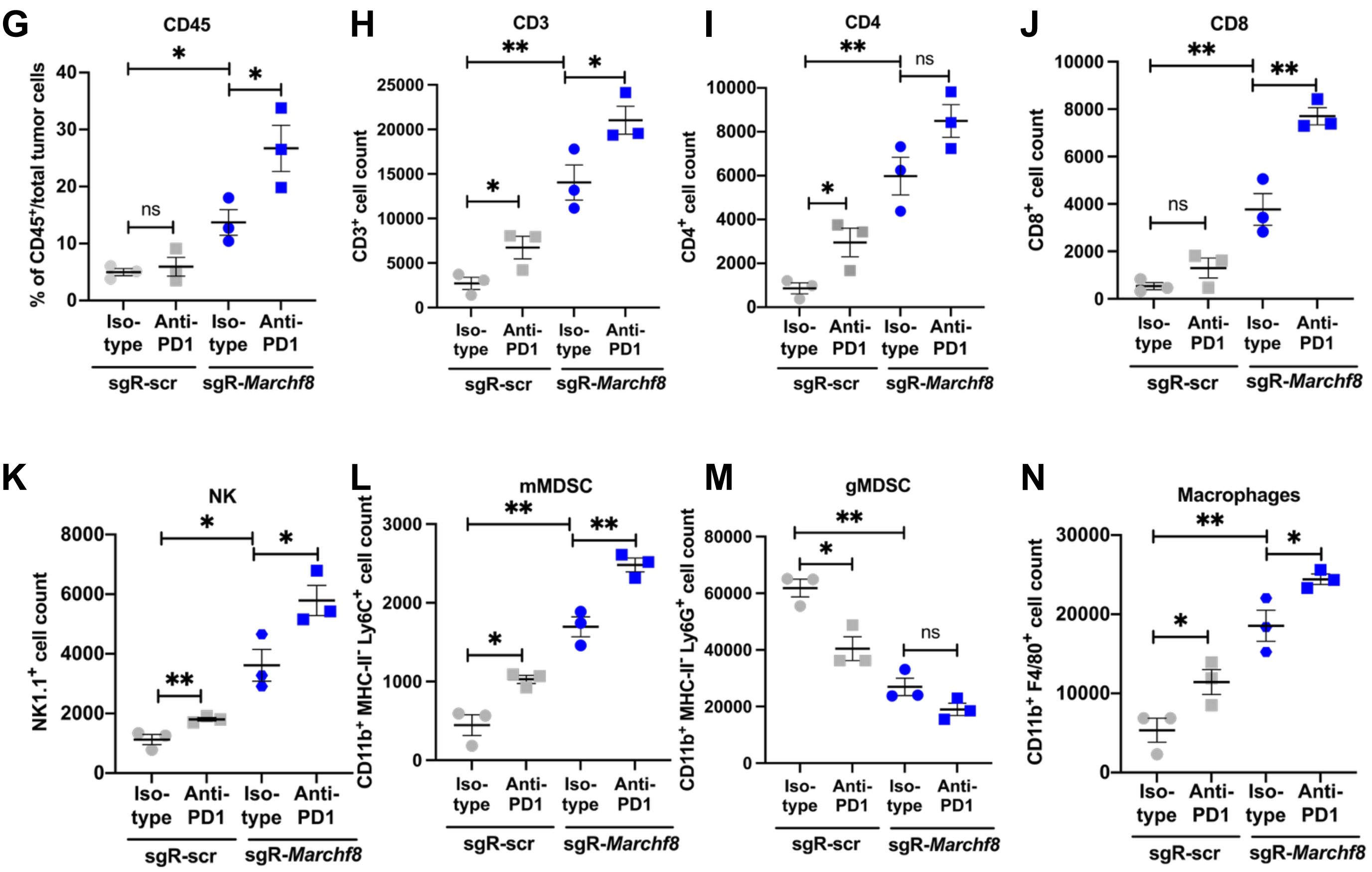

Fig. S8. Anti-PD1 treatment increases immune cell infiltration in *Marchf8* knockout-mice. C57BL/6J mice with mEERL/scr or mEERL/*Marchf8*$^{-/-}$ cells were injected with 4 doses of either rIgG2b isotype or anti-PD1 (clone #RMP1-14) antibodies as described in **Fig. 8**. Single cells isolated from tumors were stained with an antibody cocktail and analyzed by flow cytometry. Dot plots show the frequency of the CD45$^+$ cells (**A**), CD3$^+$ cells (**B**), CD4$^+$ and CD8$^+$ T cells (**C**), NK cells (**D**), mMDSC and gMDSC (**E**), and macrophages (**F**). Bar plots show the cell counts of CD45$^+$ cells (**G**), CD3$^+$ cells (**H**), CD4$^+$ T cells (**I**), CD8$^+$ T cells (**J**), NK cells (**K**), mMDSC cells (**L**), gMDSC (**M**), and Macrophages (**N**). All experiments were repeated at least three times, and the data shown are means ± SD. *P***G** values were determined by Student's *t*-test. *$p < 0.05$, **$p < 0.01$. All experiments were repeated at least three times.

Khalil et al., Fig. S9

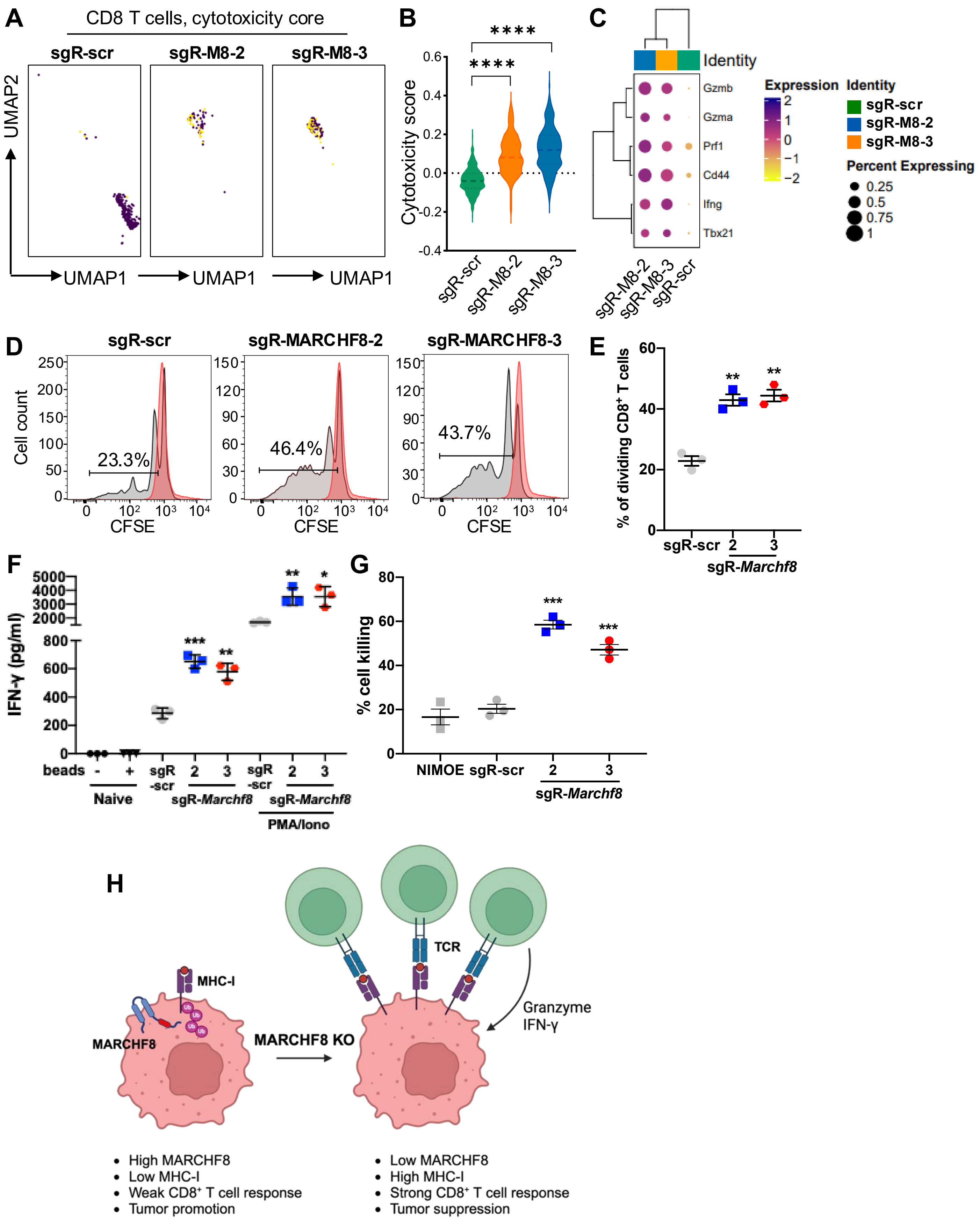

Fig. S9. *Marchf8* knockout in HPV+ HNC cells enhances cytotoxic CD8$^+$ T cell activation and tumor cell killing. UMAP plots show the cytotoxicity enrichment in CD8$^+$ T cells, the dots in yellow represent the positive in the indicated pathway (**A**), and the violin plot shows the score of the cytotoxicity enrichment in CD8$^+$ T cells (**B**). The bubble plots show the expression of the indicated genes in CD8$^+$ T cells (**C**). Splenic CD8$^+$ T cells were isolated from mice injected with mEERL cells using an EasySep mouse CD8$^+$ T cells Isolation Kit (StemCell Technologies). The isolated CD8$^+$ T cells were labeled with carboxyfluorescein succinimidyl ester (CFSE) and stimulated with anti-CD3/CD28 antibody-coated beads and recombinant mouse IL-2 and co-cultured with either mitomycin-treated mEERL/scr or mEERL/*Marchf8*$^{-/-}$ cells for 3 days. CD8$^+$ T cell proliferation was determined by dilution of CFSE intensity using flow cytometry (**D** and **E**). IFN-γ levels in the cell culture supernatant were measured by ELISA (**F**). CD8$^+$ T cell cytotoxic activity was determined by the LDH release assay using cell culture supernatant from NIMOE, or mEERL/scr or mEERL/*Marchf8*$^{-/-}$ cells co-cultured with CD8$^+$ T cells (**G**). All experiments were repeated at least three times, and the data shown are means ± SD. *P* values were determined by Student's *t*-test. $^{**}p < 0.01$, $^{***}p < 0.001$, $^{****}p < 0.0001$. (**H**) Proposed working model illustrating the enhanced tumor cell killing by CD8$^+$ T cells following *Marchf8* knockout.

Khalil et al., Fig. S10

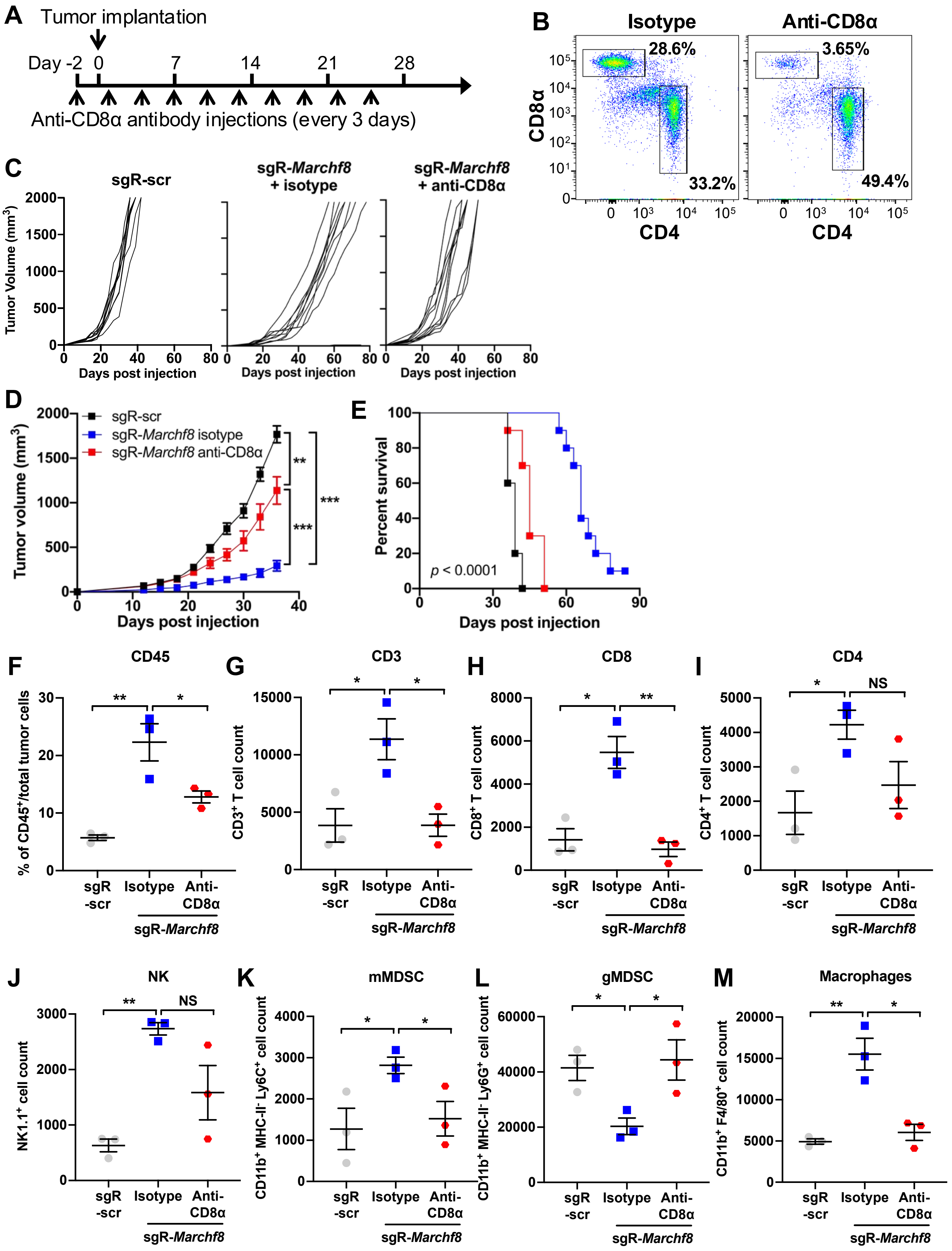

Fig. S10. CD8$^+$ T cell depletion abrogates tumor suppression by *Marchf8* knockout. C57BL/6J mice were injected with either rat IgG2b (rIgG2b) isotype or anti-mouse CD8α neutralizing (clone 2.43) antibodies. Each mouse received 10 doses of the antibody (100 μg each) starting two days before the injection of mEERL/scr or mEERL/*Marchf8*$^{-/-}$ cells (**A**). CD8$^+$ T cell depletion was validated by analyzing CD4$^+$ and CD8$^+$ T cells in splenocytes (**B**). mEERL/scr or mEERL/*Marchf8*$^{-/-}$ (sgR-*Marchf8* clone2) cells were injected into the rear right flank of C57BL/6J mice (n = 10 per group). IgG2b isotype or anti-CD8α antibodies were injected into the mice with mEERL/*Marchf8*$^{-/-}$ cells. Tumor volume was measured twice a week (**C and D**). Survival rates were analyzed using a Kaplan-Meier estimator, as described above (**E**). The data shown are means ± SD. P values were determined by two-way ANOVA analysis. $^{**}p < 0.01$, $^{***}p < 0.001$. The single cells isolated from tumors were stained with an antibody cocktail and analyzed by flow cytometry. Dot plots show the frequency of the CD45$^+$ cells (**F**), cell counts of CD3$^+$ cells (**G**), CD4$^+$ (**H**), and CD8$^+$ (**I**) T cells, NK cells (**J**), mMDSC (**K**), gMDSC (**L**), and macrophages (**M**). All experiments were repeated at least three times, and the data shown are means ± SD. P values were determined by Student's t-test. $^{*}p < 0.05$, $^{**}p < 0.01$.
